# Roles of Rufy3 in experimental subarachnoid hemorrhage-induced early brain injury via accelerating neuronal axon repair and synaptic plasticity

**DOI:** 10.1186/s13041-022-00919-6

**Published:** 2022-04-23

**Authors:** Yang Wang, Jianguo Xu, Wanchun You, Haitao Shen, Xiang Li, Zhengquan Yu, Haiying Li, Gang Chen

**Affiliations:** 1 Department of Neurosurgery & Brain and Nerve Research Laboratory, The First Affiliated Hospital of Soochow University, 188 Shizi Street, Suzhou, 215006 Jiangsu China; 2 Department of Neurosurgery, The First Affiliated Hospital of USTC, Division of Life Sciences and Medicine, University of Science and Technology of China, Hefei, Anhui China

**Keywords:** Subarachnoid hemorrhage, Rufy3, Early brain injury, Neuronal axon repair, Synaptic plasticity

## Abstract

RUN and FYVE domain-containing 3 (Rufy3) is a well-known adapter protein of a small GTPase protein family and is bound to the activated Ras family protein to maintain neuronal polarity. However, in experimental subarachnoid hemorrhage (SAH), the role of Rufy3 has not been investigated. Consequently, we aimed to investigate the potential role of Rufy3 in an in vivo model of SAH-induced early brain injury (EBI). In addition, we investigated the relevant brain-protective mechanisms. Oxyhemoglobin (OxyHb) stimulation of cultured primary neurons simulated vitro SAH condition. The SAH rat model was induced by infusing autologous blood into the optic chiasma pool and treating the rats with lentivirus-negative control 1 (LV-NC1), lentivirus-Rufy3 shRNA (LV-shRNA), lentivirus-negative control 2 (LV-NC2), lentivirus-Rufy3 (LV-Rufy3), or 8-pCPT-2′-*O*-Me-cAMP (8p-CPT) (Rap1 agonist). In experiment one, we found that the protein level of Rufy3 decreased and neuronal axon injury in the injured neurons but was rectified by LV-Rufy3 treatment. In experiment two, mRNA and protein levels of Rufy3 were downregulated in brain tissue and reached the lowest level at 24 h after SAH. In addition, the expression of Myelin Basic Protein was downregulated and that of anti-hypophosphorylated neurofilament H (N52) was upregulated after SAH. In experiments three and four, Rufy3 overexpression (LV-Rufy3) increased the interactions between Rufy3 and Rap1, the level of Rap1-GTP, and the ratio of Rap1-GTP/total GTP. In addition, LV-Rufy3 treatment inhibited axon injury and accelerated axon repair by activating the Rap1/Arap3/Rho/Fascin signaling pathway accompanied by upregulated protein expression levels of ARAP3, Rho, Fascin, and Facin. LV-Rufy3 also enhanced synaptic plasticity by activating the Rap1/MEK/ERK/synapsin I signaling pathway accompanied by upregulated protein expression levels of ERK1, p-ERK1, MEK1, p-MEK1, synaspin I, and p-synaspin I. Moreover, LV-Rufy3 also alleviated brain damage indicators, including cortical neuronal cell apoptosis and degeneration, brain edema, and cognitive impairment after SAH. However, the downregulation of Rufy3 had the opposite effect and aggravated EBI induced by SAH. Notably, the combined application of LV-Rufy3 and 8p-CPT showed a significant synergistic effect on the aforementioned parameters. Our findings suggest that enhanced Rufy3 expression may reduce EBI by inhibiting axon injury and promoting neuronal axon repair and synaptic plasticity after SAH.

## Introduction

Subarachnoid hemorrhage (SAH) is associated with high mortality and disability rates. SAH accounts for 5–7% of all cerebrovascular diseases and involves bleeding into the subarachnoid space [1, 2]. SAH often leads to the unexpected death of patients. Spontaneous SAH mostly occurs due to the rupture of intracranial aneurysms [3, 4]. Due to advancements in intracranial angiography and thorough physical examination of patients, unruptured intracranial aneurysms are increasingly being detected. Ruptured intracranial aneurysms that lead to SAH with a high Hunt and Hess grade often lead to disastrous consequences [5]. The mechanisms underlying the brain injury associated with SAH have been elucidated. Previous studies suggested that early brain injury (EBI) and cerebral angiospasm are the two main mechanisms underlying brain injury associated with SAH, and EBI is its main pathogenic factor [6, 7]. An increasing number of studies have shown that neuronal damage, microglial activation, oxidative stress, and neuroinflammation aggravate EBI [8, 9].

Human Rufy3 (RUN and FYVE domain containing 3), also known as Singar1 (single axon-related 1), is a protein that contains 469 amino acids and is highly expressed in brain tissue [10]. The crystalline structure of Rufy3 contains an RUN domain and two coiled-coil domains. The RUN domain of Rufy3 can interact with Rap2 and Rab, and is located in the N-terminal region [11]. Several proteins containing an RUN domain participate in Ras-like GTPase signaling pathways and Rab-mediated membrane trafficking [12]. In addition, Rufy3, located in the growth cone of neurons, plays a role in neuronal development by inhibiting the formation of redundant axons to maintain optimal neuronal polarity [13]. However, the pathophysiological role and associations of Rufy3 with SAH have not been explored.

Previous studies have shown that RAS-associated protein-1 (Rap1) attaches to a small GTPase of the Ras-associated protein family and regulates Rho GTPase-mediated actin cytoskeletal rearrangement and morphological changes [14, 15]. The associated Rap1 signaling pathways maintain a range of cellular functions, such as cell proliferation [16], differentiation [17], and adhesion [18]. For instance, the activation of the Rap1 signaling pathway promotes human melanoma invasion and metastasis [19]. More importantly, it has already been confirmed that Rap1 is closely related to the migration and development of neurons [20, 21].

In our study, we found that Rufy3 was located in Facsin- and Factin-enriched neuronal axons and synapses. More importantly, we found that Rufy3 overexpression alleviated post-SAH brain damage. Furthermore, our experimental results showed that Rufy3 had a positive effect on Rap1-mediated neuronal axon repair and synaptic plasticity by regulating Rufy3 expression. Hence, in brain tissue samples of experimental SAH rats and cultured primary neurons, a positive relationship between Rap1 and Rufy3 was identified, and changes in Rufy3 expression positively correlated with SAH prognosis. This was the first study to investigate the role of Rufy3 in an experimental SAH model and evaluate the relationship between Rap1 and Rufy3.

## Materials and methods

### Experimental animals

A total of 460 8-week-old clean-grade adult male SD rats (weight: 300–350 g) were provided by Suzhou Zhaoyan New Drug Research Center Co., Ltd (Suzhou, China). The rats were reared at constant temperature (18–26 °C) and humidity (40–70%). The animal experiments were authorized, approved, and supervised by the Animal Ethics Committee of the First Affiliated Hospital of Soochow University, China. The animals were raised and used in strict accordance with the National Institutes of Health guidelines.

### Neuron culture

As previously mentioned [22], we isolated and cultured primary neurons from the rat embryos on day 18. First, we separated and wiped the capillaries and meninges attached to the surface of the hemisphere. Then, the brain tissue was percussed and digested repeatedly after addition to 0.25% trypsin for 5 min. Then, the mixed liquid was centrifuged at 1000 rpm for 5 min. The deposit was added to neurobasal medium containing with 2% B27, 0.5 mM GlutaMAX TM-1, 50 U/ml penicillin and 50 U/ml streptomycin and mixed. Finally, neurons were inoculated into culture dishes, and 6-well or 12-well plates were precoated with poly-d-lysine (Sigma-Aldrich, St. Louis, MO, USA) containing fresh neurobasal medium at a density of 20,000 cells/cm_2_. The culture was kept in a 5% CO_2_ and 37 °C atmospheric incubator for 5 days. The corresponding lentivirus and enhanced infection enhancer were intervened simultaneously. Next, we incubated the neurons after oxyhemoglobin (OxyHb) stimulation under the same environment for 24 h. Finally, medium was siphoned from the plates, and primary neurons were scratched or fixed with 4% paraformaldehyde, after which relevant experiments were conducted.

### SAH model

**Fig. 1:**
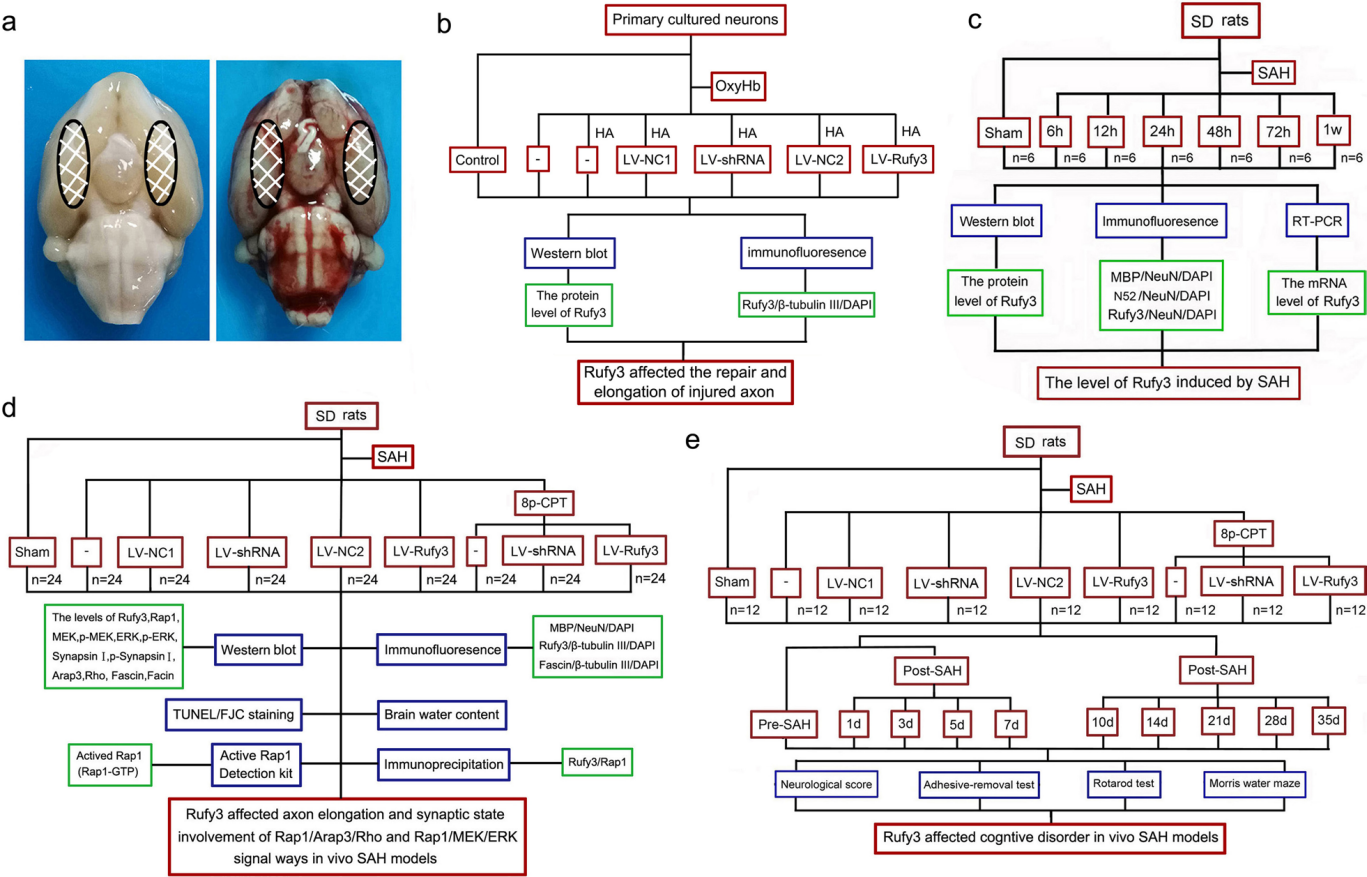
Experimental design. **a** Schematic representation of the areas selected in our study. **b** Experiment one was designed to determine the effect of Rufy3 on neuronal axons. **c** Experiment two was designed to determine the involvement of the Rufy3 in EBI under vivo SAH conditions. **d** Experiment three was designed to determine the involvement of neuroprotection through the Rufy3/Rap1 complex formation via accelerating neuronal axon repair and synaptic plasticity. **e** Experiment four was designed to evaluate the effect of Rufy3 expression on neurocognitive function

We established the SAH animal models using a stereotactic injection of autologous blood into the optic chiasm cistern, as described in our previous study [23]. After anesthesia with an intraperitoneal injection of 4% chloral hydrate (1 ml/100 g) (Sigma-Aldrich, St. Louis, MO, USA), the rats were fixed in a stereotaxic apparatus and a side needle (i.e., a needle with a round tip and a side hole located at the bottom) was stereoscopically inserted into the anterior skull base through a hole drilled in the skull. The skull was entered at the anterior midline of the sagittal point 7.5 mm away from the anterior fontanelle and the puncture direction was 45° to the coronal plane. The syringe was advanced until the tip reached the bottom of the skull and was then retracted by 0.5 mm. Then, a syringe pump was used to inject slowly 300 μl of fresh unheparinized autologous blood into the prechiasmatic cistern over 20 s. In the sham group, 300 μl of physiological saline was injected instead of blood. Twenty-four hours after SAH, 60 mL of ice-cold PBS was injected into the hearts of deeply anesthetized animals. The brain tissue of rats covered by blood clots was obtained for analysis (Fig. 1a).

### Experimental design and intervention

Experiment one was designed to explore the roles of Rufy3 in SAH in vitro. OxyHb was used to stimulate primary cultured neurons, and lentivirus-negative control 1 (LV-NC1), lentivirus-Rufy3 shRNA (LV-shRNA), lentivirus-negative control 2 (LV-NC2), and lentivirus-Rufy3 (LV-Rufy3) were used as the interventions. Cells were divided into seven groups: Control, OxyHb, OxyHb + HA, OxyHb + HA + LV-NC1, OxyHb + HA + LV-shRNA, OxyHb + HA + LV-NC2, and OxyHb + HA + LV-Rufy3 groups. The neurons received LV-NC1, LV-shRNA, LV-NC2, and LV-Rufy3 treatments at 24 h before OxyHb stimulation. The cells were lysed or fixed for western blot and immunofluorescence (IF) detection respectively. The experimental process is depicted in Fig. 1b. Experiment two was designed to determine the involvement of Rufy3 in EBI after SAH. In particular, 42 rats were assigned to seven point-in-time groups (n = 6 in each group): the sham group, the 6-, 12-, 24-, 48-, and 72-h SAH groups, or the 1-week SAH group. Brain tissue below the blood clot was obtained at different time points from SAH rats for western blot, reverse transcription-polymerase chain reaction (RT-PCR), and IF analysis. The experimental process is depicted in Fig. 1c. Experiment three was designed to examine the mechanisms underlying brain injury due to Rufy3 in EBI induced by SAH. Rats received intracerebroventricular injections of LV-NC1, LV-shRNA, LV-NC2, and LV-Rufy3 7 days before the blood injection and an intraperitoneal injection of 8-pCPT-2′-*O*-Me-cAMP (8p-CPT) 6 h after SAH. In total, 216 rats were randomly divided into nine groups (n = 24 in each): sham, SAH, SAH + LV-NC1, SAH + LV-shRNA, SAH + LV-NC2, SAH + LV-Rufy3, SAH + 8p-CPT, SAH + 8p-CPT + LV-shRNA, and SAH + 8p-CPT + LV-Rufy3 groups. First, brain tissue samples from 12 rats in each group were cut into slices for IF, Fluoro-Jade C (FJC), and terminal deoxynucleotidyl transferase-mediated dUTP nick end labeling (TUNEL) staining. Next, brain tissue samples of the remaining 12 rats in each group were euthanized, perfused, and collected for western blot assay, immunoprecipitation (IP), Rap1 activation assay, and brain edema evaluation. The experimental process is depicted in Fig. 1d. Experiment four was designed to assess the effect on cognitive and motor disorders by regulating Rufy3 expression. In total, we randomly divided 108 adult male rats into eight groups (n = 12 in each), including sham, SAH, SAH + LV-NC1, SAH + LV-shRNA, SAH + LV-NC2, SAH + LV-Rufy3, SAH + 8p-CPT, SAH + 8p-CPT + LV-shRNA, and SAH + 8p-CPT + LV-Rufy3 groups. Neurological scores, rotarod test, adhesive-removal test, and Morris water maze were conducted on rats from different groups (Fig. 1e).

### Lentiviral construction and in vivo injection

**Fig. 2:**
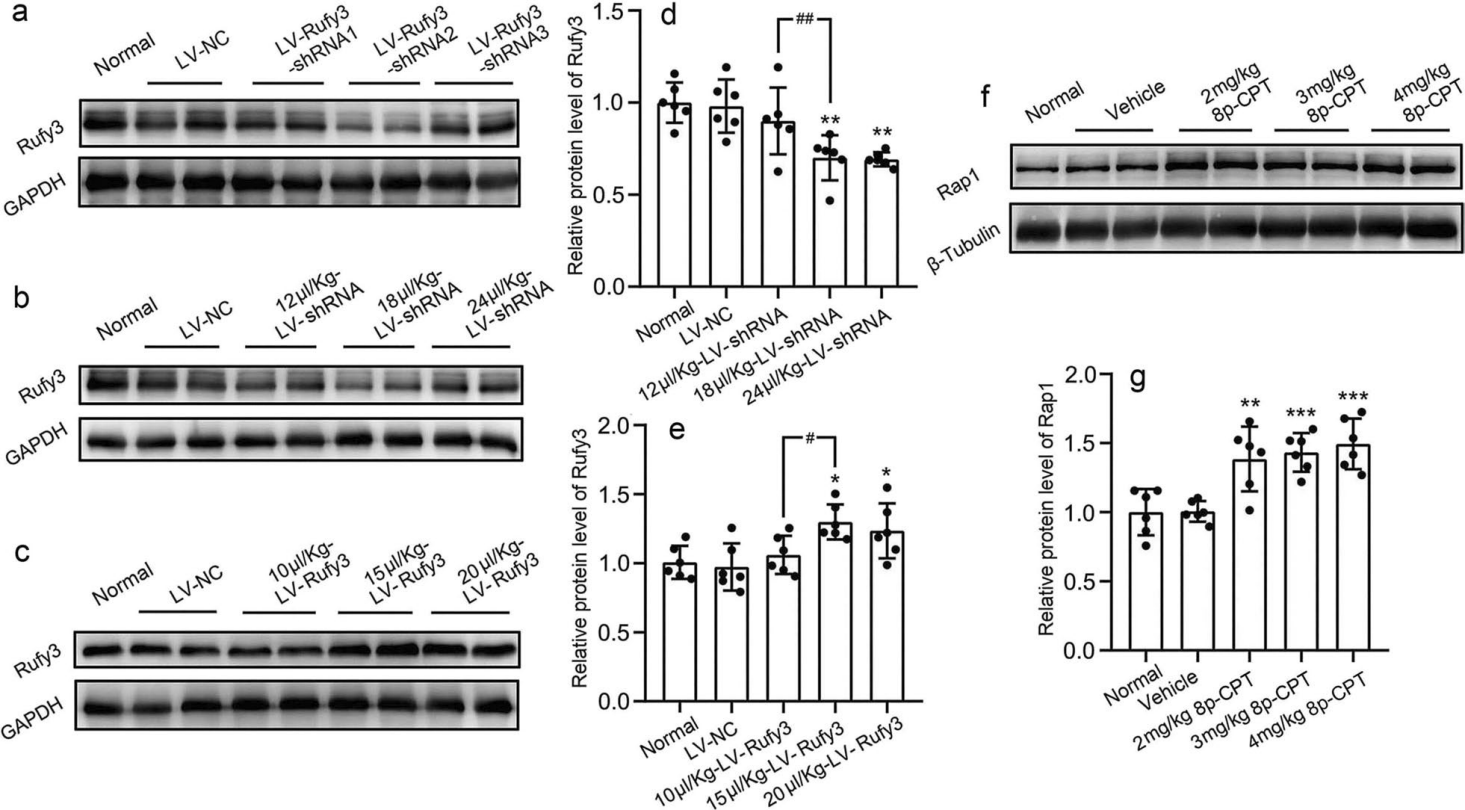
Selection of LV-Rufy3-shRNA and dose selection of LV-Rufy3-shRNA, LV-Rufy3, and 8p-CPT under normal conditions. **a** Representative bands of Rufy3 expression using three types of LV-Rufy3-shRNAs under the normal condition. LV-Rufy3-shRNA2 downregulated Rufy3 expression. **b** Representative bands of Rufy3 expression using three doses of LV-Rufy3-shRNAs (12, 18, and 24 µl/kg) under normal conditions. **c** Representative bands of Rufy3 expression using three doses of LV-Rufy3 (10, 15, and 20 µl/kg) under normal conditions. **f** Representative bands of Rap1 expression using three doses of 8p-CPT (2, 3, and 4 mg/kg) under normal conditions. **d, e, g** Quantitative analysis of Rufy3 and Rap1 expressions in different groups. The normal group was used as the standard. Data are shown as mean ± SEM (*n* = 6). *P < 0.05, **P < 0.01, ***P < 0.001 vs. LV-NC or Vehicle group; ^#^*P* < 0.05, ^##^*P* < 0.01, vs. 12 µl/kg-LV-shRNA group or 10 µl/kg-LV-Rufy3

The expression of Rufy3 was downregulated by transfection with lentiviral vectors expressing Rufy3-specific shRNA. Three lentiviral Rufy3 shRNAs (83759-1, 83580-1, and 83578-1) and a negative control virus (LVCON313, LV-NC1) were purchased from Genescript (Nanjing, China). To establish and maintain overexpression of Rufy3, a lentiviral vector of LV-Rufy3-overexpression was designed, synthesized, and constructed by Genescript. In addition, a corresponding negative control virus (LVCON55, LV-NC2) was created. The sequence elements of the lentiviral vectors were Ubi-MCS-3FLAG-CBh-gcGFP-IRES-puromycin (LV-Rufy3) and hU6-MCS-CBh-gcGFP-IRES-puromycin (LV-shRNA). The viral titers of LV-shRNA-Rufy3 and LV-Rufy3 were 4 × 10^8^ and 5 × 10^8^ TU/ml, respectively. In vitro SAH, primary neurons were transfected with the corresponding LV using 20 μl HA (HitransGA) (GeneChem) to enhance the transduction efficiency after 4 days of extraction and culture. We calculated the virus usage based on the following formula: virus volume = MOI * cell count/virus titer (multiplicity of infection [MOI] = 10). Two days after the transduction, neurons were added to OxyHb (10 μM). The experimental rat SAH model was established 7 days after lentiviral injection. The selection of LV-shRNA-Rufy3 and doses of LV-shRNA-Rufy3 and LV-Rufy3 were based on the western blot analysis in normal rats. Based on the detection of the lentivirus infection effect, we selected LV-shRNA2 as the downregulated expression of Rufy3 (Fig. 2a). In addition, 18 µl/Kg of LV-shRNA-Rufy3 (Fig. 2b, d) and 15 µl/kg of LV-Rufy3 (Fig. 2c, e) were injected into the lateral ventricles using a 10-µl Hamilton microsyringe and stereotaxic apparatus, as described in previous studies. The needle was left in place for 1 min to avoid lentivirus reverse flow after infusion [24].

### Antibodies and reagents

Anti-ARAP3 antibody (MBS9401663) was supplied by MyBioSource (San Diego, CA, USA). Anti-Rufy3 antibody (PA5-54651), anti-ERK1 antibody (13-8600), and anti-p-ERK1 antibody (PA5-94966) were purchased from Invitrogen (ThermoFisher Scientific, Waltham, MA, USA). Anti-Rho antibody (ab40673), anti-fascin antibody (ab126772), anti-facin antibody (ab205), anti-synaspin I antibody (ab64581), anti-p-synaspin I antibody (ab32532), anti-Rap1 antibody (ab113480), anti-MBP antibody (ab40390), anti-hypophosphorylated neurofilament H[N52] antibody (ab82259), anti-NeuN antibody (ab177487), anti-NeuN (ab104224), and anti-β-Tubulin III (ab41489) antibody were purchased from Abcam (Cambridge, UK). Anti-GAPDH antibodies (AF7021, T0004) and anti-β-Tubulin antibodies (DF7967, T0023) were purchased from Affinity Biosciences (Cincinnati, OH, USA). Anti-MEK1 antibody (98195S), anti-p-MEK1 antibody (26975S), anti-mouse IgG (7076S), anti-rabbit IgG (7074s), horseradish peroxidase conjugated-linked-secondary antibody, and active Rap1 Detection Kit were purchased from Cell Signaling Technology (Beverly, MA, USA). Alexa Fluor 488 (A32790) and Alexa Fluor 555 (A32794) Donkey anti-Rabbit IgG (H + L) Highly Cross-Adsorbed Secondary Antibody, and Alexa Fluor 488 (A-11001) and Alexa Fluor 555 (A-21424) Goat anti-Mouse IgG (H + L) Cross-Adsorbed Secondary Antibody were purchased from Invitrogen. Situ Cell Death Detection Kit (12156792910) and 8p-CPT (C8988), the most common Rap1 agonist, were purchased from Sigma-Aldrich, and a dose of 2 mg/kg was used to promote the expression of Rap1 based on western blot results (Fig. 2f, g).

### Western blot assay

We conducted a western blot assay 24 h after SAH, as described previously [22]. The brain samples and neurons were lysed by adding the western blot lysis buffer to phenylmethylsulfonyl fluoride. The protein concentration was determined by the bicinchoninic acid method. First, the protein samples of different groups (25 μg/lane) and molecular weight markers (8 μl/lane) were loaded on 10% and 12% sodium dodecyl-polyacrylamide electrophoresis gels. The samples were separated and electrophoretically transferred to nitrocellulose membranes. The nitrocellulose membranes were blocked with 5% skimmed milk for 1 h at room temperature and incubated with anti-ARAP3, anti-Rho, anti-Fascin, anti-Facin, anti-ERK1, anti-p-ERK1, anti-MEK1, anti-p-MEK1, anti-synaspin I, and anti-p-synaspin I antibodies at 4 °C overnight. GAPDH or β-tubulin was used as the loading control. Then, the membranes were incubated with anti-mouse IgG or anti-rabbit IgG horseradish peroxidase conjugated-linked secondary antibody (1:3000) at room temperature for 1 h. Next, the protein bands were detected using a luminescent image analyzer (Clinx ChemiScope5300, Clinx Science Instruments, Shanghai, China) after adding the developer solution to the membranes. Protein levels were analyzed using ImageJ software (National Institutes of Health, Bethesda, MD, USA) and normalized to the relative density of the normal or sham group. The ratio of phosphoprotein to total protein was used to evaluate the phosphorylation level.

### RT-PCR assay

RNA was extracted from brain tissue samples using TRIzol (ThermoFisher Scientific, 15596026) and transcribed into cDNA using the High-Capacity cDNA Reverse Transcription Kit (ThermoFisher Scientific, 4368813). The abundance of target gene RNA was detected by real-time PCR using PowerUp SYBR Green Master Mix (ThermoFisher Scientific, A25742). The results of the quantitative PCR are presented relative to the mean values of housekeeping genes (ΔΔCt method). The mRNA levels were normalized for gene expression in brain tissue samples. The Rufy3 and GAPDH primer sequences were as follows: Rufy3 Forward Primer TGCAGCCGGTCCTTAGAAATG, Rufy3 reverse Primer AGGCTAGTCTGACCCCACAG. GAPDH Forward Primer 5′-ACCCACTCCTCCACCTTTGAC-3′, GAPDH Reverse Primer 5′-TGTTGCTGTAGCCAAATTCGTT-3′.

### IF assay

IF staining was performed on cultured primary cortical neurons and brain tissue paraffin-embedded sections 24 h after SAH [22]. In experiments one and two, neurons and brain tissue specimens were fixed with 4% paraformaldehyde. Then the tissue was paraffin-embedded and sectioned into slices of 4 μm. The dewaxed sections and neurons were incubated with Rufy3 (1:200), MBP (1:300), N52 (1:300), β-tubulin III (1:250) and NeuN (1:300) antibodies overnight at 4 °C. In experiment three, the dewaxed sections were incubated with Rufy3 (1:200), Fascin (1:300), MBP (1:300) antibodies, and β-tubulin III (1:300) overnight at 4 °C. They were then incubated with the donkey anti-rabbit IgG (H + L) Highly Cross-Adsorbed Secondary Antibody or Goat anti-Mouse IgG (H + L) Cross-Adsorbed Secondary Antibody at 37 °C for 1 h. Then, the sections and neurons were washed three times on the next day. Next, the water-soluble mounting medium, 4,6-diamino-2-phenylindole (SouthernBiotech, Birmingham, AL, USA), was added to the sections for cover slipping. Finally, the fluorescence of brain regions and neurons was observed under a fluorescence microscope (OLYMPUS BX50/BX-FLA/DP70; Olympus Co., Tokyo Japan), and ImageJ software was used to quantify the fluorescence intensity.

### IP assay

IP detection was performed 24 h after SAH, as described previously [25]. First, the radioimmunoprecipitation assay lysis buffer was added to lyse the brain samples. Then, the lysate was incubated with a rabbit monoclonal antibody against Rap1 or rabbit IgG overnight at 4 °C with orbital shaking. Then, protein A + G Sepharose beads were added to every immunocomplex. Simultaneously, the pyrolysis mixture was incubated at 4 °C for 4 h with orbital shaking. Ultimately, the immunoblotting method was used to isolate and detect the protein.

### Rap1 activation assay

An Active Rap1 Detection Kit was used for the accurate determination of the level of activated Rap1 [26]. In brief, brain tissue samples were lysed on ice and the obtained lysis solution was mixed and incubated with glutathione agarose beads coupled to GST-RalGDS (Ral guanine dissociation stimulator) to detect activated Rap1 levels. After washing the beads, the samples were loaded onto SDS-polyacrylamide gels, separated, and electrophoretically transferred to the nitrocellulose membranes. The membranes were probed with anti-Rap1 antibody after blocking in 3% bovine serum albumin (BSA) for 1 h at 4 °C overnight. Then, the membranes were incubated with an anti-rabbit IgG horseradish peroxidase (HRP) conjugate. Total Rap1 levels were measured using brain tissue sample lysates that were not subjected to coincubation with beads. Immunoreactive proteins were visualized by enhanced chemiluminescence using a luminescent image analyzer (Clinx ChemiScope 5300, Clinx Science Instruments), and protein intensities were analyzed using Image J software.

### Measurement of neuronal axon lengths

To measure the lengths of neuronal axons, β-tubulin III positive neurite lengths were measured as previously described [27]. Briefly, six random microscopic fields (× 400 magnification) were chosen and photographed under a fluorescent microscope. All β-tubulin III immunofluoresce-positive cells in the microscopic field were selected to measure neuronal axon length. Neuronal axon length was defined as the distance between the body and the farthest tip of the neurite and the measurement of neuronal axon length was performed by the ImageJ software (National Institutes of Health, Bethesda, MD, USA).

### Brain edema

As mentioned previously, wet and dry weighing methods were used to assess the cerebral edema index at 48 h after SAH [28]. We collected fresh brain tissue and promptly weighed it to record the wet weight. Next, the sample was dried at 100 °C for 24 h, and weighed to calculate the dry weight. The cerebral edema index was calculated as ([wet weight − dry weight]/wet weight) × 100%.

### TUNEL staining

We used the TUNEL method to evaluate cortical cell apoptosis using the In Situ Cell Death Detection Kit at 24 h after SAH [29]. Briefly, we incubated rat brain sections with 0.1% Triton X-100 for 8 min. After washing the sections three times, we added the sections to the TUNEL reaction mixture at 37 °C for 1 h. Then, 4,6-diamino-2-phenylindole was added to cover the brain sections after washing them with PBST. Finally, we observed the sections under a fluorescence microscope. To estimate the degree of cortical cell apoptosis, the ratio of TUNEL-positive cells (red fluorescence) was recorded as the apoptotic index for each section. In brief, the TUNEL-positive cells were counted by an observer who was blinded to the sham and experimental groups. The apoptotic index was defined as the average number of TUNEL positive cells in each section counted in six microscopic fields (× 400 magnification).

### FJC staining

FJC, a highly specific and sensitive fluorescent dye used to mark neuronal degradation [30], was added to the sections at 24 h after SAH. Briefly, brain sections were dewaxed and soaked in 0.06% KMnO_4_ solution at room temperature for 15 min in the dark. The sections were incubated with FJC working solution and 0.1% acetic acid solvent for 1 h. The sections were air-dried at room temperature and sealed with a neutral balsam medium. Finally, three sections of each rat and six microscope fields in each tissue section were examined under a fluorescence microscope and photographed to count the FJC-positive cells.

### Neurological scoring

We used an 18-point scoring system (Table 1) to evaluate the neurologic function of rats at 24 h after SAH.

**Table 1:** Neurological evaluation of rats post SAH

Test	Score			
	0	1	2	3
Spontaneous activity (in cage for 5 min)	No movement	Barely moves position	Moves but does not approach at least three sides of cage	Moves and approaches at least three sides of cage
Spontaneous movements of all limbs	No movement	Slight movement of limbs	Moves all limbs but slowly	Move all limbs same as pre-SAH
Movements of forelimbs (outstretching while held by tail)	No outreaching	Slight outreaching	Outreach is limited and less than pre-SAH	Outreach same as pre-SAH
Climbing wall of wire cage		Fails to climb	Climbs weakly	Normal climbing
Reaction to touch on both side of trunk		No response	Weak response	Normal response
Response to vibrissae touch		No response	Weak response	Normal response

### Adhesive removal test

The adhesive removal test was used to assess sensory and motor coordination abilities after SAH [30]. First, the rats were placed in a glass box and a circular sticker was placed on the palm of each forepaw of the rats. The time taken by the rats to remove all the stickers was recorded. The rats were regularly trained daily for 3 days before the test. The test was conducted 1 day before modeling and on days 1, 3, 5, 7, 10, 14, 21, 28, and 35 after SAH.

### Rotarod test

This test was used to evaluate the movement ability of rats by rotating a cylinder provided by Anhui Zhenghua Biological Equipment Co. Ltd (Hefei, Anhui, China) [30]. The rats were placed on a horizontal axis that had been set at a stationary rate of 4 to 30 r/min for 1 min. Notably, the test was terminated immediately once the rats fell to the ground or gripped the device for two cycles. The duration spent by the rats on the horizontal axis was recorded. As with the adhesive removal test, the rats were trained for 3 days prior to modeling. The test was also conducted 1 day before modeling and on days 1, 3, 5, 7, 10, 14, 21, 28, and 35 after SAH.

### Morris water maze

The Morris water maze experiment has been described previously [31]. The Morris water maze device consisted of a circular pool with a diameter of 2 m and a height of 0.75 m. The circular pool was filled with water mixed with melanin at a depth of 0.4 m at an appropriate temperature. Four equidistant points were randomly designated as North (N), South (S), East (E), and West (W) to divide the pool into four equivalent quadrants (NW, NE, SE, and SW). The circular platform had a diameter of 30 cm. We placed a 10 × 10 cm transparent plexiglass platform at the confluence of the eight random equidistant lines (N, S, E, W, NW, NE, SE, and SW), 2 cm below the surface of the water. A vidicon was installed on the ceiling above the swimming pool and used to track the trajectories of the animals. The rats in the experimental groups were trained for 4 consecutive days before formal testing. The trial lasted for 60 s and the per-interval testing time of each rat was 5 min. If the rat arrived at the platform within 60 s, it would rest on the platform for 15 s. In contrast, if the rat did not arrive at the platform within 60 s, it would be guided to the platform. The time and distance required for the rat to enter the water to find the underwater concealed platform and stand on it were recorded as water maze latency and swimming distance. The Morris water maze tests were conducted one day prior to modeling and on days 10, 14, 21, 28, and 35 after SAH, simultaneously, their latency and swimming distance were recorded.

### Statistical analysis

The data were analyzed using the GraphPad Prism 7.0 software (San Diego, CA, USA). Data are expressed as the mean ± SEM (standard error of the mean). One-way or two-way analysis of variance (ANOVA) was used for multiple comparisons, and Bonferroni’s or Tukey’s post hoc test was used for comparisons between two pairs in multiple groups. *P* < 0.05 was considered statistically significant.

## Results

### The mRNA and protein levels of Rufy3 were downregulated and neuronal axons were damaged after SAH

**Fig. 3:**
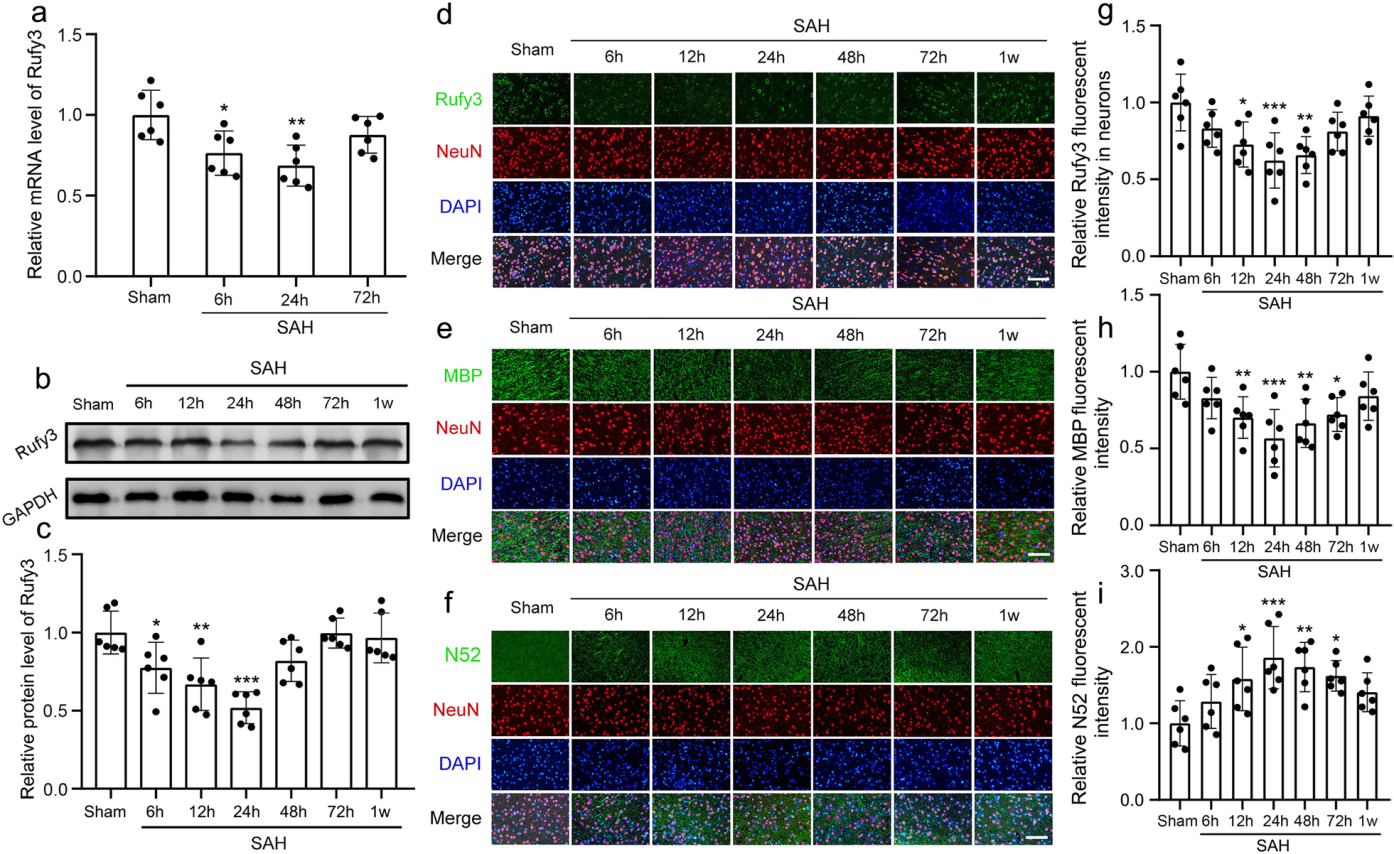
Rufy3 mRNA and protein expression levels and neuronal axon damage following SAH. **a** Quantitative analysis of Rufy3 mRNA level. The sham group was used as a control. **b** Representative band of Rufy3 detected by western blot. **c** Quantitative analysis of Rufy3 at different stages following SAH. The sham group was used as a control. **d**–**f** Double immunofluorescence of Rufy3, MBP, and N52 (green, Alexa Fluor 488) and neuronal marker (NeuN; red, Alexa Fluor 555), and Rufy3 mainly located in the neurons. Nuclei were stained with DAPI (blue). Scale bars = 100 μm. **g**–**i** Immunopositivity of Rufy3, MBP, and N52 in neurons. The Sham group was used as the standard. Data are shown as mean ± SEM (*n* = 6). *P < 0.05, **P < 0.01, ***P < 0.01, vs. Sham group

Western blotting, IF staining, and RT-PCR were performed to detect the changes in Rufy3 expression after SAH. The results of PCR showed decreased Rufy3 mRNA level of SAH 6 h and SAH 24 h groups compared to the sham group (*P* < 0.05 and *P* < 0.01, respectively; Fig. 3a). The western blot results showed that the Rufy3 expression level significantly decreased after SAH, and was the lowest at 24 h after SAH, followed by gradual recovery within 1 week (*P* < 0.05, *P* < 0.01, and *P* < 0.001, respectively; Fig. 3b, c). In addition, the IF results showed that Rufy3 immunopositivity was decreased after SAH (12 h, 24 h and 48 h) compared to the sham group (*P* < 0.05, *P* < 0.01, and *P* < 0.001, respectively; Fig. 3d, g). We also found evidence of neuronal axon damage after SAH in terms of decreased immunopositivity to MBP (12 h, 24 h, 48 h, and 72 h) (*P* < 0.05, *P* < 0.01, and *P* < 0.001, respectively; Fig. 3e, h) and increased immunopositivity to N52 (12 h, 24 h, 48 h, and 72 h) (*P* < 0.05, *P* < 0.01, and *P* < 0.001, respectively; Fig. 3f, i). These results suggested that Rufy3 participates in the pathological process underlying EBI, and is inhibited after SAH. Furthermore, we found that the ideal time point for further interventions in this study was 24 h after SAH.

### Silencing/overexpressing Rufy3 inhibited/accelerated Rufy3 expression and neuronal axon repair after SAH in vivo and in vitro

**Fig. 4:**
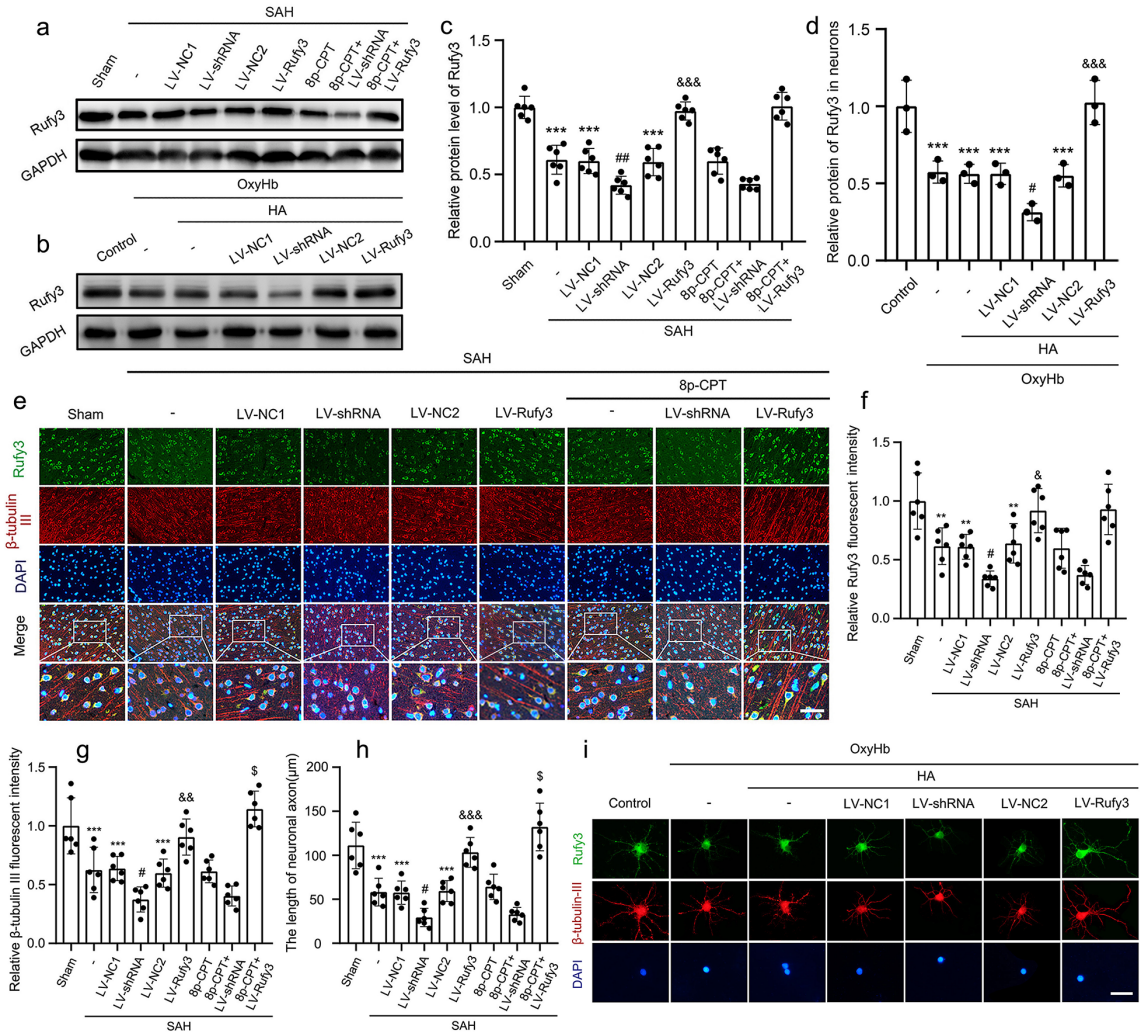
The protein expression levels of Rufy3 and the state of neuronal axon under LV-shRNA and LV-Rufy3 treatments after vivo and vitro SAH. **a** Representative bands of Rufy3 detected by western blot under 8p-CPT, LV-shRNA and LV-Rufy3 treatments following vivo SAH. **b** Representative bands of Rufy3 detected by western blot under LV-shRNA and LV-Rufy3 treatments following vitro SAH. **c**, **d** Quantitative analysis of Rufy3 in different groups following vivo and vitro SAH. The sham and control group were used as a control. **e** Double immunofluorescence analysis of Rufy3 (green, Alexa Fluor 488) and β-tubulin III (axon; red, Alexa Fluor 555); nuclei were stained with DAPI (blue). Scale bars = 32 μm. **f, g** Quantitative fluorescent intensity analysis of Rufy3 and β-tubulin III expressions in different groups. The sham group was used as the standard. **h** Quantitative analysis of the length of neuronal axon in different groups. **i** Double immunofluorescence of Rufy3 (green, Alexa Fluor 488) and β-tubulin III (axon; red, Alexa Fluor 555). Nuclei were stained with DAPI (blue). Scale bars = 100 μm. Data are shown as mean ± SEM (*n* = 6). **P < 0.01, **P < 0.001 vs. Sham group; *P < 0.001 vs. Control group; ^#^*P* < 0.05, ^##^*P* < 0.01 vs. LV-NC1 groups; ^&^*P* < 0.05, ^&&^*P* < 0.01, ^&&&^*P* < 0.001 vs. LV-NC2 group; ^$^*P* < 0.05 vs. LV-Rufy3 group

Western blotting was used to assess the target protein expression in brain tissue and neurons after treatment with LV-shRNA and LV-Rufy3. In the in *vivo* SAH model (Fig. 4a, c), the western blot results showed that the expression levels of Rufy3 in the SAH, LV-NC1, and LV-NC2 groups were lower than those in the sham group (*P* < 0.001). LV-shRNA treatment resulted in significantly lower Rufy3 protein levels than the LV-NC1 group (*P* < 0.01). Conversely, LV-Rufy3 treatment increased the reduced Rufy3 expression level after SAH (*P* < 0.001). However, 8p-CPT (a Rap1 agonist) had no significant effect on Rufy3 expression. In vitro SAH model (Fig. 4b, d), we found that the expression levels of Rufy3 in the OxyHb, OxyHb + HA, OxyHb + HA (LV-NC1 and LV-NC2) groups were all lower than that in the control group (*P* < 0.001). LV-shRNA treatment further decreased the reduced Rufy3 expression level (*P* < 0.05), but LV-Rufy3 treatment increased the reduced Rufy3 expression level after OxyHb intervention (*P* < 0.001). Double immunofluorescence Rufy3 and β-tubulin III IF staining was used to evaluate the relation of Rufy3 expression and neuronal axon injury. In vivo SAH model (Fig. 4e–h), the immunofluorescent staining results showed significantly shorter axon lengths and neuronal axon injury in the SAH, LV-NC1, and LV-NC2 groups than in the control group (*P* < 0.01 and *P* < 0.001). Neuronal axon injury was further aggravated by LV-shRNA treatment after SAH (*P* < 0.05), while LV-Rufy3 treatment resulted in significant repair of the injured axons and the elongation of axons compared to the LV-NC2 group (*P* < 0.05, *P* < 0.01, and *P* < 0.001). Finally, the combined use of 8p-CPT and LV-Rufy3 enhanced the repair of injured axons compared to the use of 8p-CPT or LV-Rufy3 treatment alone (*P* < 0.05). In vitro SAH model (Fig. 4i), we observed that the growth of neurites was inhibited and that the length of the axon was shortened under the OxyHb stimulation. LV-shRNA treatment aggravated neuronal axon injury and inhibited axon elongation, conversely, LV-Rufy3 decelerated neuronal axon injury and accelerated axon repair. In conclusion, neuronal axon length was positively correlated with the expression of Rufy3 both in vivo and in vitro in the SAH model.

### Silencing/overexpressing Rufy3 inhibited/accelerated Rufy3/Rap1 complex formation and the level of Rap1-GTP

**Fig. 5:**
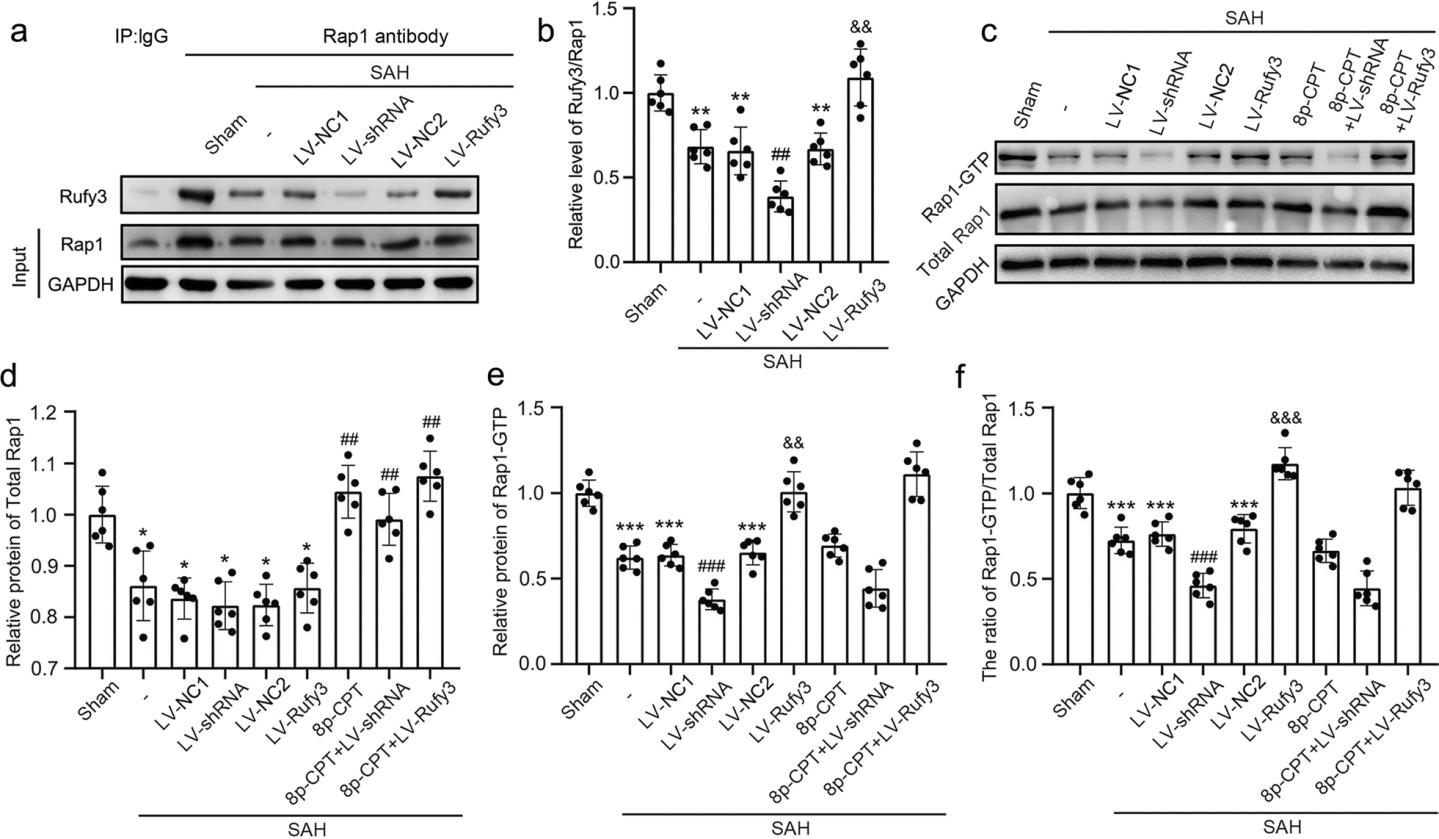
Interaction between Rufy3/Rap1 and protein expression levels of Rufy3, and activated Rap1 under LV-shRNA and LV-Rufy3 treatments after SAH. **a** Sample lysates with Rap1 antibody (IgG was used as a negative control) and Rufy3 were measured by immunoprecipitation. **b** Quantitative analysis of Rufy3/Rap1 under LV-shRNA and LV-Rufy3 treatments at 24 h after SAH. The sham group was used as a control. **c** Representative bands of Rap1-GTP and total Rap1 were detected by western blot. **d**–**f** Quantitative analysis of Rap1-GTP, total Rap1, and the ratio of Rap1-GTP/total Rap1 in different groups following SAH. The sham group was used as a control. *P < 0.05, **P < 0.01, **P < 0.001 vs. Sham group; ^#^*P* < 0.05, ^##^*P* < 0.01, ^###^*P* < 0.001 vs. LV-NC1 groups; ^&&^*P* < 0.01, ^&&&^*P* < 0.001 vs. LV-NC2 group

IP was used to detect Rufy3/Rap1 complex formation (Fig. 5a, b) and Rap1-GTP pull-down was used to determine Rap1-GTP (the activated form of Rap1) levels (Fig. 4c–f). Rufy3/Rap1 complex formation was significantly reduced after SAH due to reduced Rufy3 expression (*P* < 0.01). However, LV-shRNA further inhibited Rufy3/Rap1 complex formation compared to the LV-NC1 group (*P* < 0.01). In contrast, LV-Rufy3 increased Rufy3/Rap1 complex formation compared to the LV-NC2 group (*P* < 0.01). In the SAH, SAH + LV-NC1, SAH + LV-shRNA, SAH + LV-NC2 and SAH + LV-Rufy3 groups, the total Rap1 level was lower than that in the sham group (*P* < 0.05), whereas the addition of 8p-CPT significantly upregulated the total Rap1 level (*P* < 0.01). In contrast, the Rap1-GTP level and the ratio of Rap1-GTP/total Rap1 were significantly decreased after SAH, whereas LV-shRNA significantly decreased and LV-Rufy3 significantly increased the Rap1-GTP level and Rap1-GTP/total Rap1 (*P* < 0.01 and *P* < 0.001, respectively).

### Silencing/overexpressing Rufy3 inhibited/accelerated neuronal axon repair by suppressing/activating the Rap1/Arap3/Rho/Fascin signaling axis after experimental SAH

**Fig. 6:**
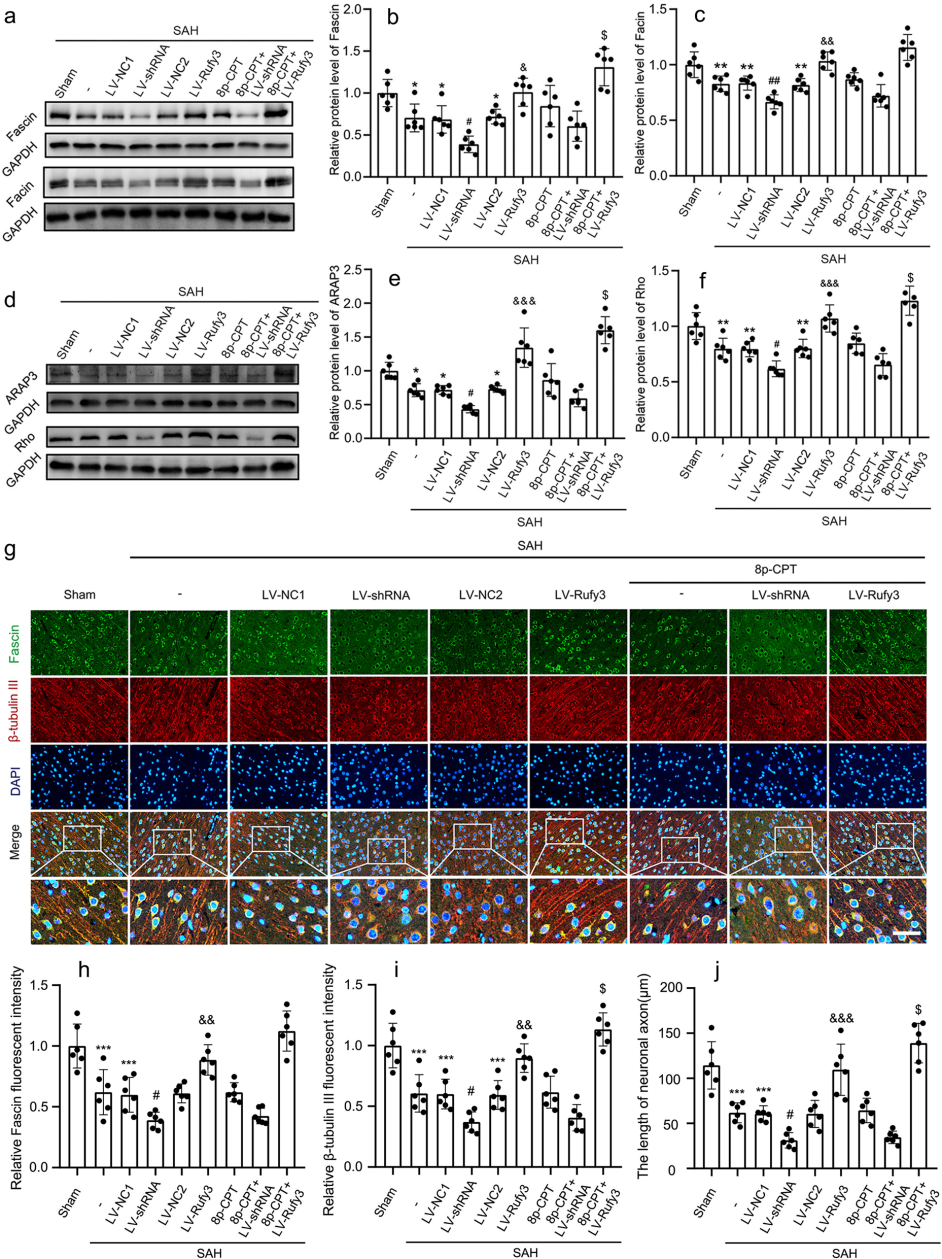
Effects of LV-shRNA and LV-Rufy3 on the Rap1/Arap3/Rho/Fascin signaling axis after experimental SAH. **a** Representative bands of Fascin and Facin expressions. **b, c** Quantitative analysis of Fascin and Facin. The sham group was used as control. **d** Representative bands of ARAP3 and Rho expressions. **e**, **f** Quantitative analysis of ARAP3 and Rho. The sham group was used as control. **g** Double immunofluorescence of Fascin (green, Alexa Fluor 488) and β-tubulin III (axon; red, Alexa Fluor 555); nuclei were stained with DAPI (blue). Scale bars = 40 μm. **h, i** Quantitative fluorescent intensity analysis of Rufy3 and β-tubulin III expressions in different groups. The sham group was used as the standard. **j** Quantitative analysis of the length of neuronal axons in different groups. *P < 0.05, **P < 0.01, ***P < 0.001 vs. Sham group; ^#^*P* < 0.05, ^##^*P* < 0.01 vs. LV-NC1 groups; ^&^*P* < 0.05, ^&&^*P* < 0.01, ^&&&^*P* < 0.001 vs. LV-NC2 group; ^$^*P* < 0.05 vs. LV-Rufy3 group

Western blotting was used to assess target protein expression in brain tissue in the Rap1/Arap3/Rho/Fascin signaling pathway after treatment with LV-NC1, LV-shRNA, LV-NC2, LV-Rufy3, and 8p-CPT (Fig. 6a–f). The results of western blot analysis showed that the expression levels of ARAP3, Rho, Fascin, and Facin in the SAH, SAH + LV-NC1, and SAH + LV-NC2 groups were significantly lower than those in the sham group (*P* < 0.05 and *P* < 0.01). LV-Rufy3 treatment resulted in significantly higher protein levels of ARAP3, Rho, Fascin, and Facin than those in the LV-NC2 group *(P* < 0.05, *P* < 0.01, and *P* < 0.001). In contrast, the expression levels of ARAP3, Rho, Fascin, and Facin in the LV-shRNA groups were further decreased compared to those in the LV-NC1 group (*P* < 0.05 and *P* < 0.01). Notably, the combined application of 8p-CPT and LV-Rufy3 improved the reduced ARAP3, Rho, Fascin, and Facin expression levels compared to 8p-CPT or LV-Rufy3 treatment alone after SAH (*P* < 0.05). Moreover, Fascin and β-tubulin III IF staining were used to evaluate neuronal axon injury and repair (Fig. 6g–j). Similar trends were observed for Fascin and β-tubulin III after SAH, suggesting neuronal axon injury in the SAH, LV-NC1, and LV-NC2 groups (*P* < 0.001). LV-shRNA treatment aggravated the neuronal axon injury after SAH (*P* < 0.05), while LV-Rufy3 treatment resulted in significant repair of the injured axons compared to the LV-NC2 group (*P* < 0.01 and *P* < 0.001). Finally, the combined use of 8p-CPT and LV-Rufy3 was superior for the repair of injured axons compared to the use of 8p-CPT or LV-Rufy3 treatment alone after SAH (*P* < 0.05). In conclusion, neuronal axon length was positively correlated with the expression of Fascin both in vivo and in vitro in the SAH model.

### Silencing/overexpressing Rufy3 inhibited/accelerated synaptic plasticity by suppressing/activating the Rap1/MEK/ERK/Synapsin I signaling axis after experimental SAH

**Fig. 7:**
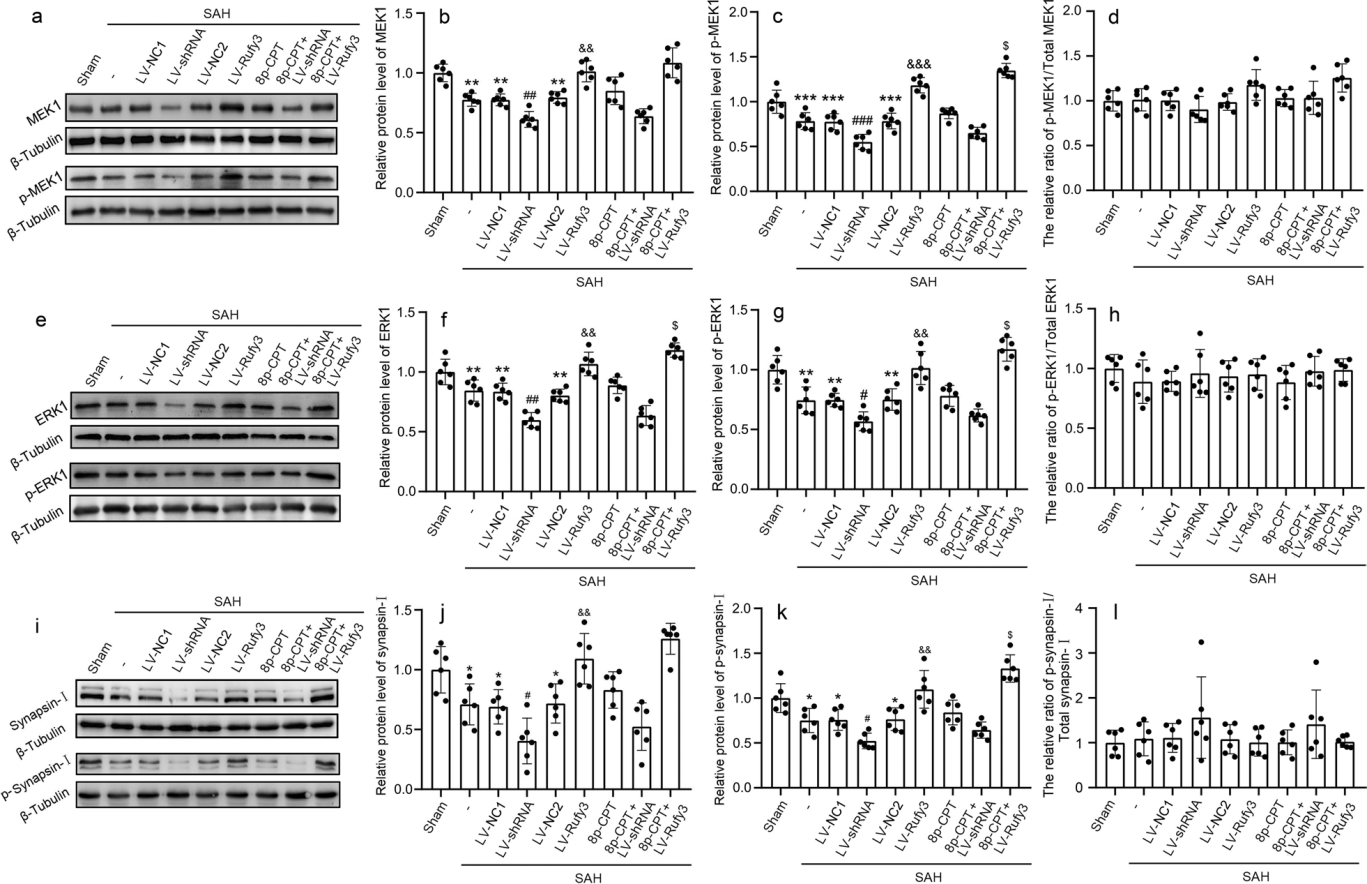
Effects of LV-shRNA and LV-Rufy3 on the Rap1/MEK/ERK/Synapsin I signaling axis after experimental SAH. **a** Representative bands of MEK1 and p-MEK1 expressions. **b**–**d** Quantitative analysis of MEK1, p-MEK1 and the ratio of p-MEK1/total MEK1. The sham group was used as control. **e** Representative bands of ERK1, and p-ERK1 expressions. **f**–**h** Quantitative analysis of ERK1, p-ERK1 and the ratio of p-ERK1/total MEK1. The sham group was used as control. **i** Representative bands of synapsin I and p-synapsin I expressions. **j**-**l** Quantitative analysis of synapsin I, p-synapsin I and the ratio of p-synapsin I/total synapsin I. The sham group was used as control. *P < 0.05, **P < 0.01, ***P < 0.001 vs. Sham group; ^#^*P* < 0.05, ^##^*P* < 0.01, ^###^*P* < 0.001 vs. LV-NC1 groups; ^&^*P* < 0.05, ^&&^*P* < 0.01, ^&&&^*P* < 0.001 vs. LV-NC2 group; ^$^*P* < 0.05 vs. LV-Rufy3 group

Western blotting was used to assess the target protein expression levels in brain tissue in the Rap1/MEK/ERK/Synapsin I signaling axis after treatment with LV-NC1, LV-shRNA, LV-NC2, LV-Rufy3, and 8p-CPT (Fig. 7a–l). The western blot results showed that the expression levels of ERK1, p-ERK1, MEK, p-MEK, synaspin I, and p-synaspin I in SAH, LV-NC1, and LV-NC2 groups were significantly lower than those in the sham group (*P* < 0.05, *P* < 0.01, and *P* < 0.001). LV-Rufy3 treatment resulted in significantly higher protein levels of ERK1, p-ERK1, MEK1, p-MEK1, synaspin I, and p-synaspin I than those in the LV-NC2 treatment (*P* < 0.05 and *P* < 0.01). In contrast, the expression levels of ERK, p-ERK, MEK, p-MEK, synaspin I, and p-synaspin I in the LV-shRNA groups were significantly decreased compared to those in the LV-NC1 group (*P* < 0.05, *P* < 0.01, and *P* < 0.001). Notably, the combined use of 8p-CPT and LV-Rufy3 improved the reduced ERK, p-ERK, MEK, p-MEK, synaspin I, and p-synaspin I expression levels compared to the use of 8p-CPT or LV-Rufy3 alone after SAH (*P* < 0.05). It is noteworthy that there were no significant differences among the ratios of p-MEK1/total MEK1, p-ERK1/total ERK1 and p-synaspin I/total synaspin I in different groups.

### Silenced Rufy3 increased and overexpressed Rufy3 decreased SAH-induced brain edema, neuronal apoptosis and degradation, and axonal injury

**Fig. 8:**
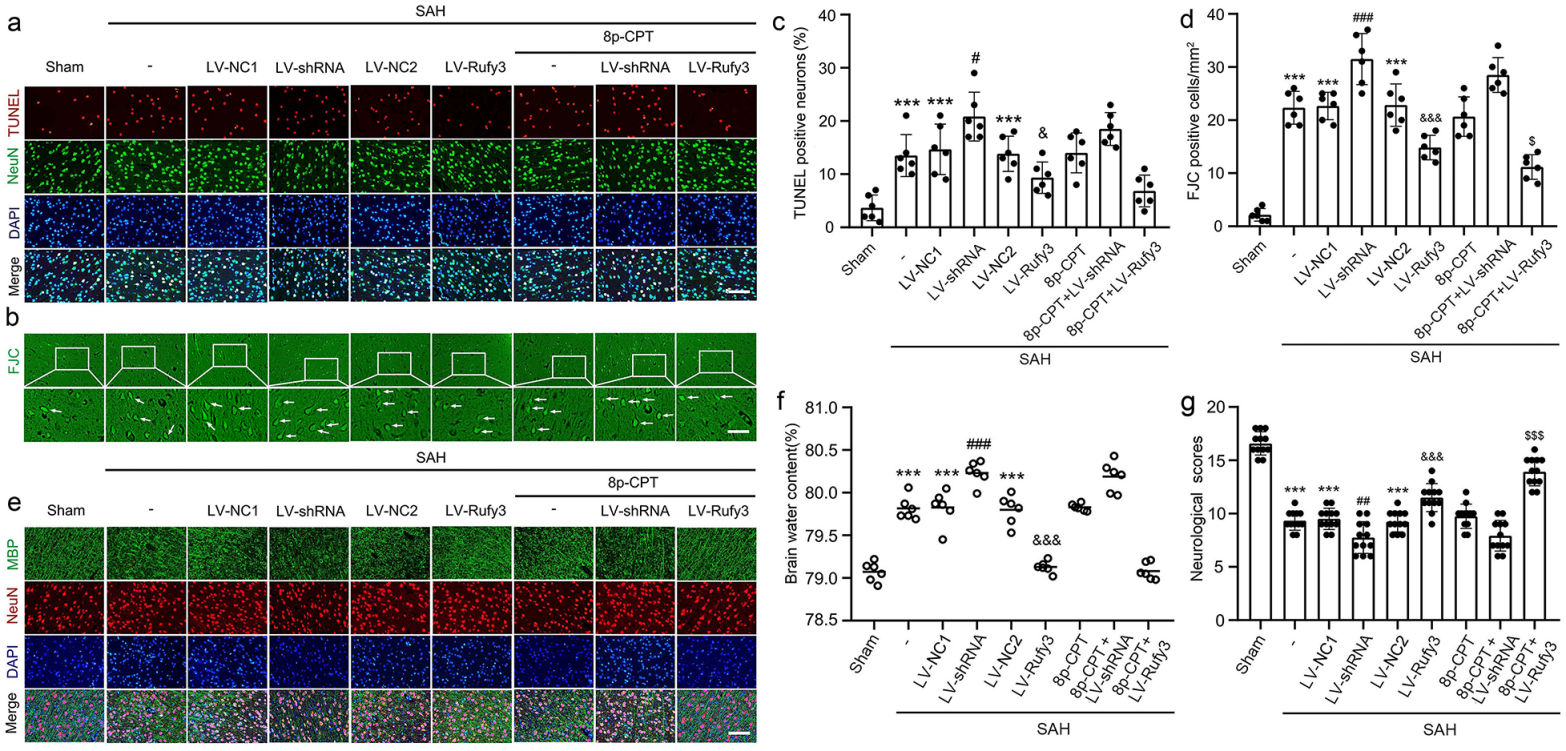
Effect of LV-shRNA and LV-Rufy3 on cortical cell apoptosis and degradation, brain edema, and neurological score after SAH. **a** Double immunofluorescence analysis of TUNEL staining (red, Alexa Fluor 555) and neuronal marker (NeuN; green, Alexa Fluor 488) was performed to assess neuronal apoptosis at 24 h after SAH. **b** Fluoro-Jade C staining (green) was performed to evaluate neuronal degeneration and arrows pointed to FJC-positive cells. **c** Quantitative analysis of apoptotic neuron percentage. **d** Quantitative analysis of Fluoro-Jade C positive cells/mm^2^ in brain sections in each group. **e** Double immunofluorescence of MBP (green, Alexa Fluor 488) and neuronal marker (NeuN; red, Alexa Fluor 555), and Rufy3 mainly located in the neurons. **f** Brain water content. **g** Neurological scoring. Scale bars = 100 μm. ***P < 0.001 vs. Sham group; ^#^*P* < 0.05, ^##^*P* < 0.01, ^###^*P* < 0.001 vs. LV-NC1 groups; ^&^*P* < 0.05, ^&&&^*P* < 0.001 vs. LV-NC2 group; ^$^*P* < 0.05, ^$$$^*P* < 0.001 *vs*. LV-Rufy3 group

To investigate the effects of Rufy3 on SAH-induced EBI, neuronal apoptosis and degradation were evaluated using TUNEL (Fig. 8a, c) and FJC staining (Fig. 8b, d). In addition, neuronal axon injury was assessed using MBP IF staining (Fig. 8e). Compared to the sham group, the proportion of TUNEL- and FJC-positive cells and damaged axons in brain tissues from the SAH, LV-NC1, and LV-NC2 groups were significantly increased (*P* < 0.001). LV-shRNA treatment further increased the proportion of TUNEL- and FJC-positive cells and damaged axons compared to the LV-NC1 group (*P* < 0.05 and *P* < 0.001). In addition, FJC- and TUNEL-positive cells and damaged axons were significantly decreased after LV-Rufy3 treatment compared to the LV-NC2 groups (*P* < 0.05 and *P* < 0.001). Finally, the combined use of 8p-CPT and LV-Rufy3 played a synergistic role in decreasing the proportions of TUNEL- and FJC-positive cells and damaged axons in brain tissues after SAH (*P* < 0.05). We assessed the degree of SAH-induced cerebral edema by the dry–wet method (Fig. 8f). The SAH, LV-NC1, and LV-NC2 groups showed a higher edema index than the sham group (*P* < 0.001). The degree of brain edema was significantly increased after LV-shRNA treatment (*P* < 0.001) but reduced after LV-Rufy3 treatment (*P* < 0.001). No significant statistical differences were observed in the brain edema index among the SAH, LV-NC1, and LV-NC2 groups.

### Silencing Rufy3 aggravated, while overexpressing Rufy3 improved, SAH-induced neurological function deficits after SAH

**Fig. 9:**
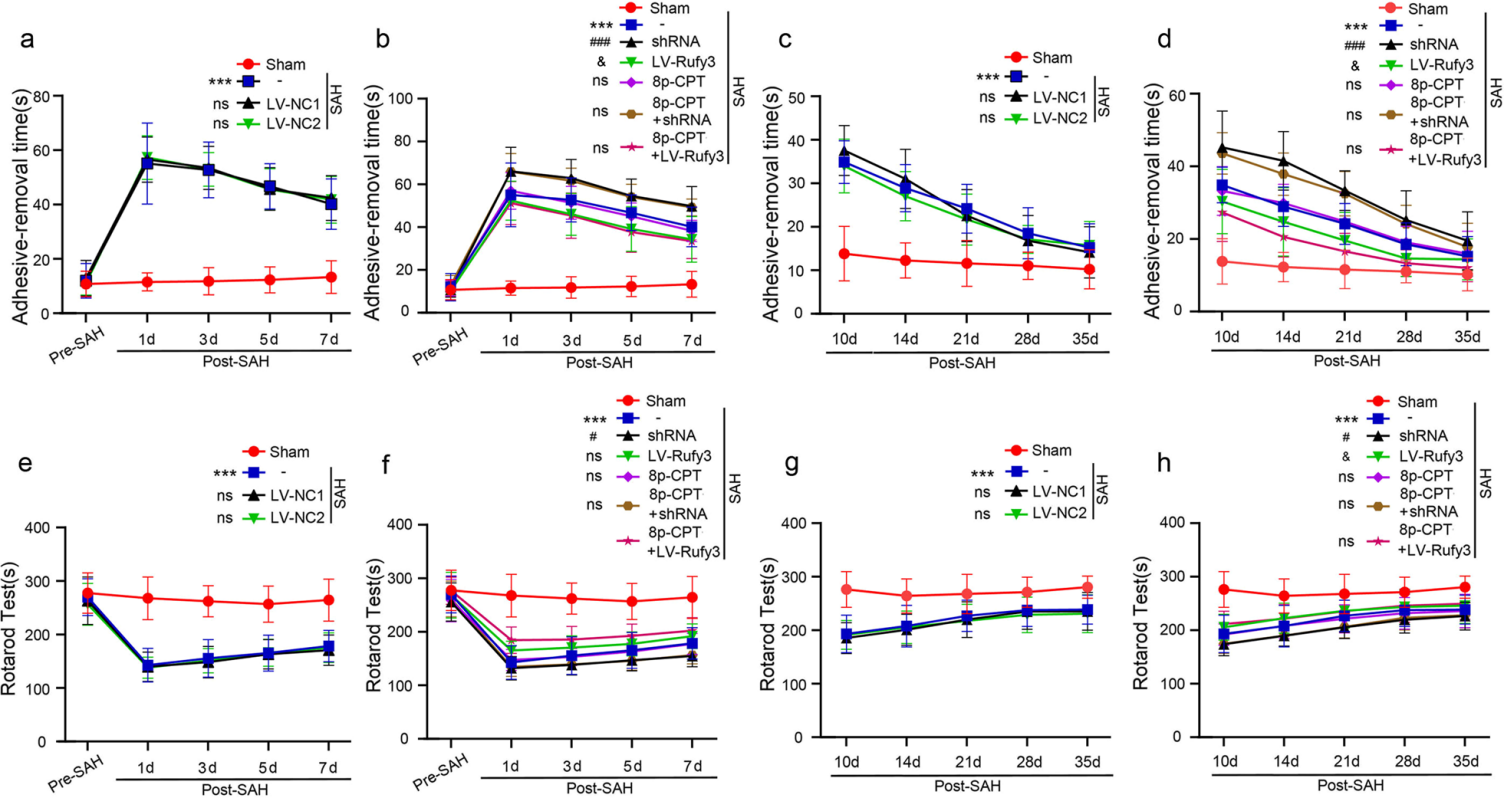
Effect of silenced/overexpressed Rufy3 on the recovery of sensory and locomotor function of rats after SAH. **a**–**d** Adhesive removal test. Silencing Rufy3 delayed, while overexpressing Rufy3 accelerated, the recovery of sensory function in rats after SAH. **e**–**h** Rotarod test. Silencing Rufy3 delayed, while overexpressing Rufy3 accelerated, the recovery of locomotor function in rats after SAH. Data are shown as the mean ± SEM (n = 12). ***P < 0.01, vs. Sham group; ^#^*P* < 0.05, ^###^*P* < 0.001, vs. LV-NC1 group, ^&^*P* < 0.05, ^&&&^*P* < 0.001 vs. LV-NC2 group

**Fig. 10:**
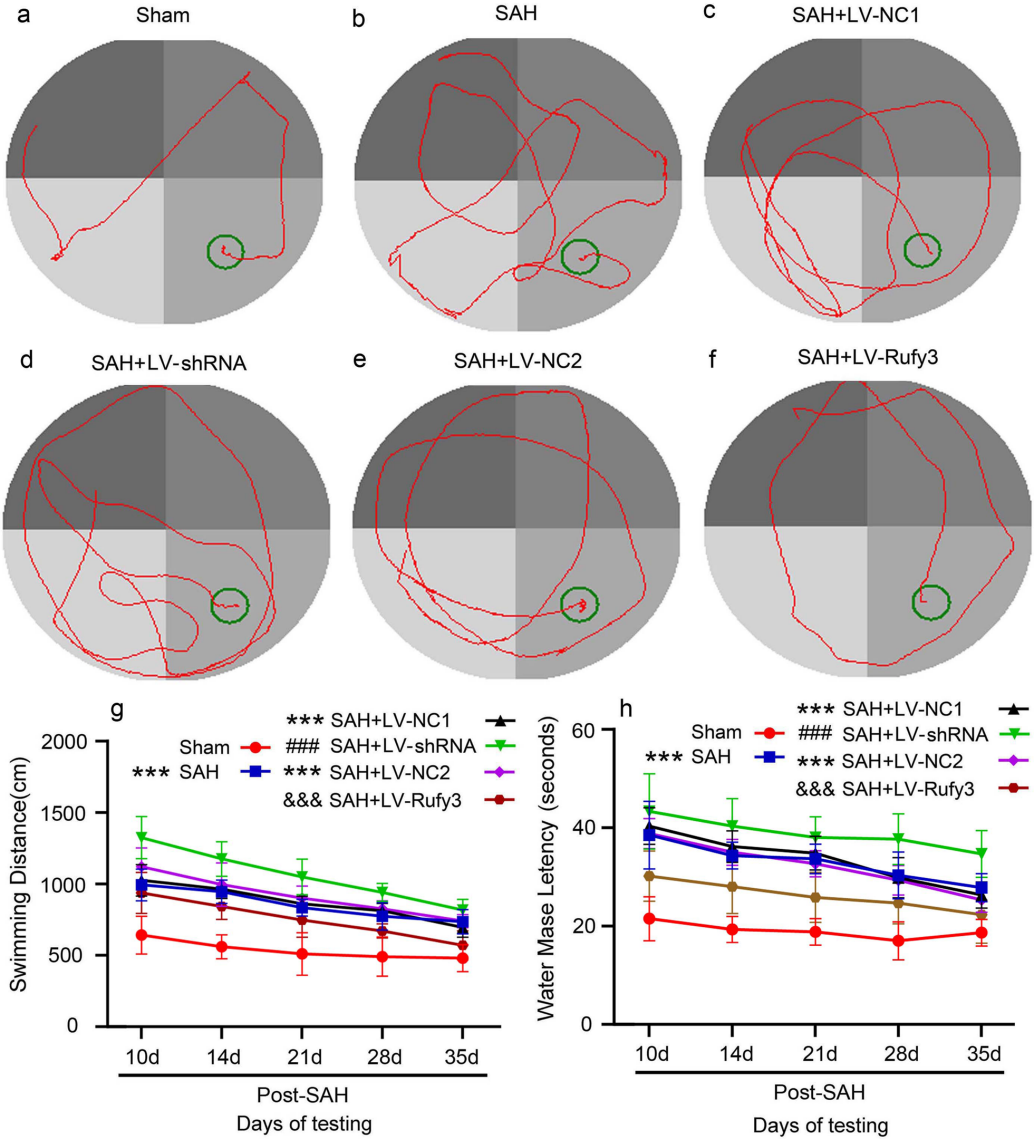
Effect of silenced/overexpressed Rufy3 on the recovery of spatial and motor learning ability of rats after SAH. **a**–**f** Swimming tracks (red) in the Morris water maze (MWM). Silencing Rufy3 had a longer trajectory, whereas overexpressing Rufy3 had a shorter trajectory. The green circle indicates the target platform. **g** Swimming distance in the MWM. Silencing Rufy3 increased the swimming distance, whereas overexpressing Rufy3 decreased the swimming distance. **h** Escape latency in the MWM. Silencing Rufy3 increased the escape latency, whereas overexpressing Rufy3 decreased the escape latency. Data are shown as mean ± SEM (n = 10). ***P < 0.01, vs. Sham group; ^###^*P* < 0.001, vs. LV-NC1 group; ^&&&^*P* < 0.001, vs. LV-NC2 group

To investigate whether Rufy3 overexpression improves neurological function, the behavioral activity of all rats was measured by behavioral scoring. As shown in Fig. 8g, the rats showed severe neurological impairment after SAH (*P* < 0.001) compared to the sham group, which was significantly alleviated by LV-Rufy3 (*P* < 0.001) and further aggravated by LV-shRNA (*P* < 0.01). The neurological impairment was dramatically improved after the combined use of 8p-CPT and LV-Rufy3 (*P* < 0.001). We evaluated the effect of Rufy3 modulation on the sensory functions of rats. Over the short- (1–7 days) and long- (10–35 days) periods after SAH, we conducted a debonding test to evaluate forelimb coordination and sensorimotor function (Fig. 9a–d). After SAH, the rats took a long time to remove the stickers after SAH (*P* < 0.001). However, when Rufy3 was reduced, this time duration was significantly increased (*P* < 0.001). In contrast, when Rufy3 was increased, this time duration was significantly shortened (*P* < 0.001). Finally, the combined use of 8p-CPT and LV-Rufy3 significantly shortened this duration compared to the isolated use of 8p-CPT or LV-Rufy3 (*P* < 0.05). In addition, we evaluated the locomotor function of rats using the rotarod test (Fig. 9e–h). The locomotor ability of rats was significantly reduced after SAH (*P* < 0.001). Rufy3 silencing further aggravated locomotor ability dysfunction (*P* < 0.05), whereas Rufy3 overexpression accelerated the recovery of locomotor ability (*P* < 0.05). We investigated the spatial and motor learning abilities of rats following SAH using the Morris water maze test. The representative trajectories in different groups are shown in Fig. 10a–f. In the Morris water maze test (Fig. 10g, h), the escape latency in the SAH group was significantly increased (*P* < 0.001). Interestingly, the escape latencies in the LV-shRNA group were more prolonged than those in the LV-NC1 group over the long-term period after SAH (*P* < 0.001), whereas the escape latencies in the LV-Rufy3 group were significantly reduced over the long-term period after SAH (*P* < 0.001) compared to those in the LV-NC2 group. The swimming distance in the SAH, LV-NC1, and LV-NC2 groups was also significantly increased compared to that in the sham group (*P* < 0.001). We found that over the long-term period after SAH, the swimming distance in the LV-shRNA group was increased compared to that in the LV-NC1 group (*P* < 0.001), whereas the swimming distance in the LV-Rufy3 group was significantly decreased compared to the LV-NC2 group (*P* < 0.001). In summary, no apparent differences in the escape latency or swimming distance were observed among the SAH, LV-NC1, and LV-NC2 groups.

## Discussion

The results of experiment one showed that the protein level of Rufy3 decreased and neuronal axon injury in the injured primary neurons was induced by OxyHb, while it was further aggravated by LV-shRNA treatment and rectified by LV-Rufy3 treatment. The results of experiment two confirmed that Rufy3 was associated with EBI and neuronal axon damage induced by SAH. The most significant decrease in Rufy3 mRNA and protein levels was observed at 24 h after SAH. In addition, we observed that Rufy3 was highly expressed in neurons and positively correlated with neuronal axon damage. In addition, Rufy3 was accompanied by decreased MBP and increased N52 IF intensity. In experiments three and four, we found that the interaction of Rufy3 and Rap1 significantly decreased after SAH. Intracerebroventricular infusions of LV-shRNA reduced the Rufy3/Rap1 complex formation and the level of Rap1-GTP. Overexpression of Rufy3 accelerated neuronal axon repair by activating the Rap1/Arap3/Rho/Fascin signaling axis and synaptic plasticity by activating Rap1/MEK/ERK/Synapsin I signal axis after experimental SAH. In addition, LV-Rufy3 treatment alleviated EBI and provided effective neuroprotection by alleviating SAH-induced neuronal apoptosis and degradation, brain edema, and impaired neurological behavior and cognitive function. In contrast, LV-shRNA treatment had the an opposite effect and distinctly aggravated EBI after SAH. Ultimately, the combined use of 8p-CPT and LV-Rufy3 played a synergistic role in alleviating EBI and provided effective neuroprotection by the above mechanisms in brain tissues after SAH. Based on the results of these experiments, we concluded that the interaction of Rufy3 and Rap1 contributed to EBI by accelerating neuronal axon repair and synaptic plasticity after SAH. Few studies have evaluated axon injury, remodeling, and related internal regulatory mechanisms after SAH. However, some studies have shown that Rufy3 plays a significant role in maintaining the growth and development of neurons, cell polarity, and axon elongation and extension [32]. Therefore, the present study mainly focused on how Rufy3 regulates neuronal axon repair and synaptic plasticity and provides an effective treatment for post-SAH neurological dysfunction.

After aneurysm rupture, arterial blood flows into the subarachnoid space at a high speed, which leads to biomechanical damage to the peripheral brain tissue, possibly leading to demyelination and neuronal axon injury [33]. In addition, after SAH, blood in the subarachnoid space may cause mechanical compression of deep white matter due to a sharp increase in intracranial pressure [34]. Hence, early decompressive craniectomy has positive effects on the motor and cognitive outcomes of severe SAH patients [35]. Although several pathological lesions, such as oxidative stress, neuroinflammation, and blood–brain barrier destruction, may lead to neuronal injury [36–38], the exact molecular mechanisms remain unclear. Professor Costantino Iadecola pointed out that the brain has extraordinary self-protection ability, and the brain tissue has started its own endogenous protection mechanism to defend against extrinsic injury, simultaneously, another important theory was emphasized that the treatment measures that fully mobilize the endogenous brain protection mechanism were expected to achieve an ideal neuroprotection effect [39]. In this study, neuronal axon injury became unavoidable after SAH and it was essential that the endogenous protection system was activated as soon as possible. Therefore, Rufy3 overexpression accelerated neuronal axon repair after experimental SAH.

Rufy3 is expressed in the developing mouse brain neurons and is a new member of the family of Factin-associated proteins, which are important in the formation of nerve axons. Rufy3 downregulation results in axon shortening and an increased proportion of neurons with multiple axons. In contrast, Rufy3 upregulation increases axon length and neuronal maturation [13, 40]. In addition, Rufy3 interacts with bundle protein and brain developmental protein (Drebrin) and colocalizes with Factin in hippocampal neurons in the peripheral and transition regions of the axonal growth cone. Rufy3 silencing or downregulation disrupts the normal distribution of Fascin and Factin [32], which was confirmed in an experimental SAH model that showed that the expressions levels of post-SAH Fascin and Factin were significantly decreased and positively correlated with Rufy3 expression.

Rap1 is a small GTPase from the Ras family of GTPases, which is activated by upstream signaling factors, including cytokines, growth factors, and chemokines [14]. Rap1 is considered the central regulator of cell adhesion and motility [18], and the asymmetric distribution of activated Rap1 promotes cell polarity and migration via remodeling of the actin cytoskeleton at the leading edge of the cells [41]. Previous studies have shown that Rap1 activity, especially that of activated Rap1, plays a crucial role in maintaining normal human breast epithelial cell polarity, while increased aberrant activation of Rap1 leads to tumor formation and malignancy progression [42]. In addition, Rap1 accelerates vascular endothelial growth factor receptor 2 activation and angiogenesis through the expression of integrins [43]. Rap1 regulates the recycling and affinity of integrins, and integrin activation is related to the actin cytoskeleton either through their polarized spatial distribution or cytoskeleton dynamics [15]. Rap1 is closely related to nervous system development, for instance, Rap1 induced and oriented the radial migration of multipolar neurons in the developing neocortex [20]. Yang et al. also found that Drosophila Rap1 played an important role in controlling intersegmental nerve b motor axon guidance during neural development and contributed to axonal growth and guidance through gain-of-function studies [21]. No previous studies have been conducted on Rap1 related to neuronal axon growth and synaptic plasticity in secondary brain injury. Nevertheless, Rap1 overexpression by the use of an agonist did not alleviate neuronal axon and synaptic plasticity injury or neurological function impairment. We also found that Rap1 participated in the formation of the Rufy3/Rap1 complex, which plays an important role in activating the Rap1/Arap3/Rho/Fascin and Rap1/MEK/ERK/Synapsin I signaling axes after experimental SAH.

**Fig. 11:**
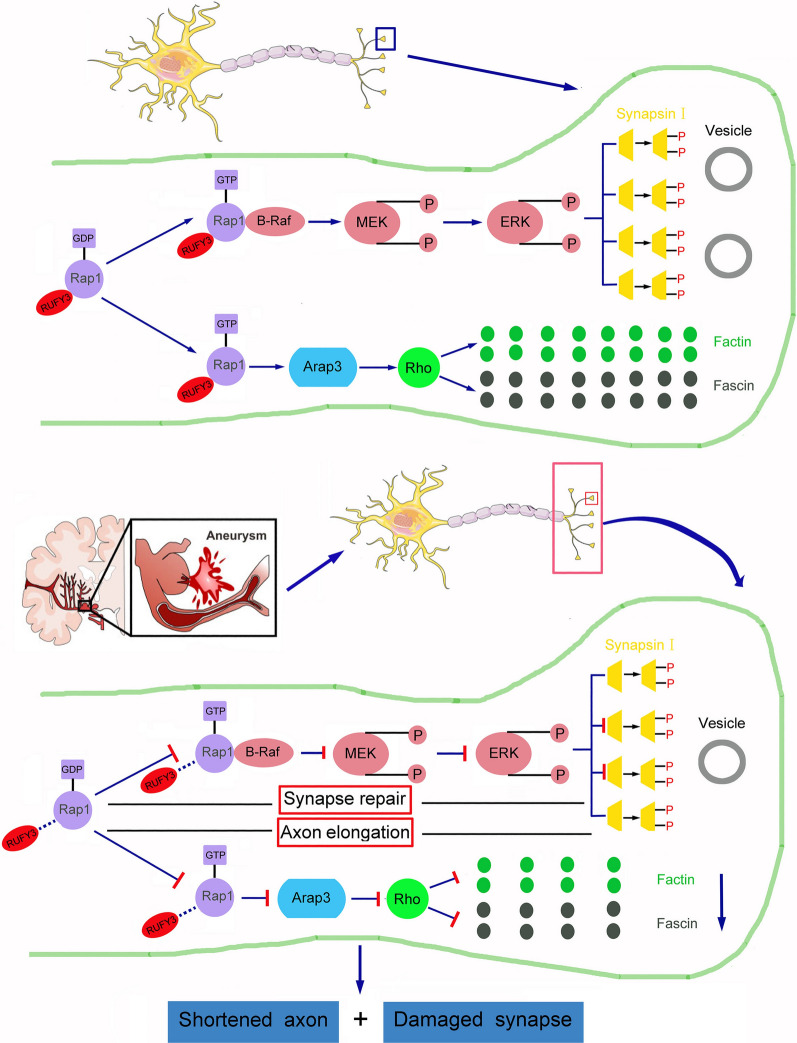
Schematic diagram of the potential neuroprotective mechanisms of Rufy3 after SAH. Following SAH, the inhibition of Rufy3 expression resulted in an increase in neuronal axon and synaptic damage accompanied by hindered Rufy3/Rap1 complex formation. In addition, Rufy3 overexpression contributed to Rufy3/Rap1 complex formation and accelerated neuronal axon repair via activating the Rap1/Arap3/Rho/Fascin signaling axis. It also accelerated synaptic plasticity by activating the Rap1/MEK/ERK/Synapsin I signaling axis after experimental SAH

Previous research on Rufy3 in tumors mainly focused on gastrointestinal tumors. A gastric cancer study found that Rufy3 promoted gastric cancer cell invasion and migration by causing the formation of Factin-enriched protrusive structures in the cell periphery [44]. In the present study, Rufy3 overexpression promoted neuronal axon repair and synaptic plasticity in the experimental SAH rat models resulting in high expression of Fascin, Facin, and synapsin I inside the proximal axons of neurons, and activation of the Rap1/Arap3/Rho/Fascin and Rap1/MEK/ERK/Synapsin I signaling axes. Consequently, the combined regulation of Rufy3 and Rap1 expression may significantly reduce axonal injury after SAH and improve the prognosis of patients with SAH (Fig. 11).

The present study had several limitations. First, we did not assess the role of Rufy3/Rap1 complex in the subunit junction. In addition, it was not possible to accurately evaluate neuronal axon repair and synaptic plasticity using IF, and electron microscope scanning and Golgi staining may be more suitable and accurate. In conclusion, our study provides comprehensive evidence supporting the role of the Rufy3/Rap1 complex in experimental SAH rat models and neuroprotection. First, the combined use of 8p-CPT (Rap1 agonist) and LV-Rufy3 activated both the Rap1/Arap3/Rho/Fascin signaling axis and the Rap1/MEK/ERK/Synapsin I signaling axis and ameliorated brain injury indicators, including brain edema, cortical neuron apoptosis and degradation, and neurological defects via accelerating Rufy3/Rap1 complex formation induced by SAH. LV-shRNA treatment had the opposite effect and aggravated brain damage via inhibiting Rufy3/Rap1 complex formation. The results of these experiments confirmed that the neuroprotective effect of the Rufy3/Rap1 complex may result from the acceleration of neuronal axon repair and synaptic plasticity after SAH. Therefore, the Rufy3/Rap1 complex may be a therapeutic target for hemorrhagic stroke.

## Data Availability

All data in the current study are available from the corresponding author on reasonable request.
