# Correction to: Roles of Rufy3 in experimental subarachnoid hemorrhage-induced early brain injury via accelerating neuronal axon repair and synaptic plasticity

**DOI:** 10.1186/s13041-024-01170-x

**Published:** 2025-01-14

**Authors:** Yang Wang, Jianguo Xu, Wanchun You, Haitao Shen, Xiang Li, Zhengquan Yu, Haiying Li, Gang Chen

**Affiliations:** 1 Department of Neurosurgery & Brain and Nerve Research Laboratory, The First Affiliated Hospital of Soochow University, 188 Shizi Street, Suzhou, Jiangsu 215006 China; 2 Department of Neurosurgery, The First Affiliated Hospital of USTC, Division of Life Sciences and Medicine, University of Science and Technology of China, Hefei, Anhui China

**Correction to: Mol Brain 15, 35 (2022).**

10.1186/s13041-022-00919-6

Following publication of the original article [1], the authors identified two errors in the Figs. 4e and 8b. Specifically, the amplification area of SAH + LV-Rufy3 group in the Fig. 4e and the amplification area of SAH + LV-NC1 group in the Fig. 8b were incorrect. The other elements of the figure remain unchanged.

In addition, there are two mistakes in the Figure caption of Figs. 4 and 6. Specifically, the description of β-tubulin III (NeuN; red, Alexa Fluor 555) should instead read as β-tubulin III (axon; red, Alexa Fluor 555).

These changes do not affect the results or conclusions of this study.

The authors apologize for any inconvenience caused.

The incorrect and correct Figs. 4, 6 and 8 are indicated hereafter.

The incorrect Fig. 4 (caption):

**Fig. 4:**
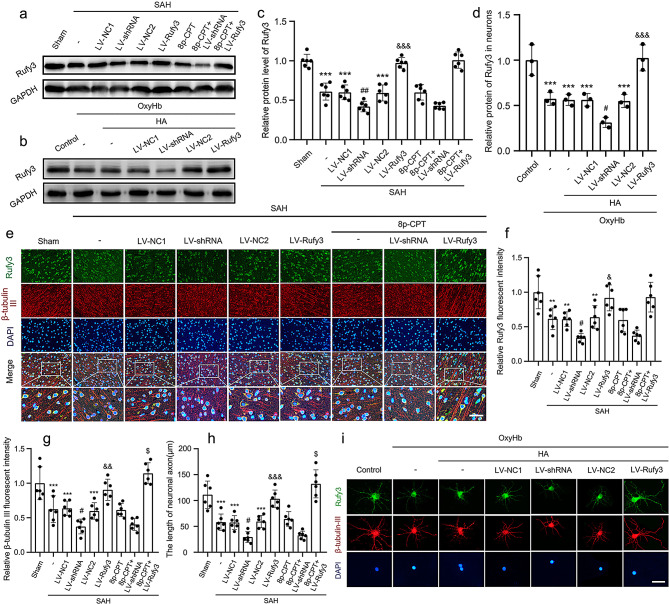
The protein expression levels of Rufy3 and the state of neuronal axon under LV-shRNA and LV-Rufy3 treatments after vivo and vitro SAH. **a** Representative bands of Rufy3 detected by western blot under 8p-CPT, LV-shRNA and LV-Rufy3 treatments following vivo SAH. **b** Representative bands of Rufy3 detected by western blot under LV-shRNA and LV-Rufy3 treatments following vitro SAH. **c**, **d** Quantitative analysis of Rufy3 in different groups following vivo and vitro SAH. The sham and control group were used as a control. e Double immunofluorescence analysis of Rufy3 (green, Alexa Fluor 488) and β-tubulin III (NeuN; red, Alexa Fluor 555); nuclei were stained with DAPI (blue). Scale bars = 32 μm. **f**, **g** Quantitative fluorescent intensity analysis of Rufy3 and β-tubulin III expressions in different groups. The sham group was used as the standard. **h** Quantitative analysis of the length of neuronal axon in different groups. i Double immunofluorescence of Rufy3 (green, Alexa Fluor 488) and β-tubulin III (axon; red, Alexa Fluor 555). Nuclei were stained with DAPI (blue). Scale bars = 100 μm. Data are shown as mean ± SEM (*n* = 6). ^**^P < 0.01, ^**^P < 0.001 vs. Sham group; ^*^P < 0.001 vs. Control group; ^#^*P* < 0.05, ^##^*P* < 0.01 vs. LV-NC1 groups; ^&^*P* < 0.05, ^&&^*P* < 0.01, ^&&&^*P* < 0.001 vs. LV-NC2 group; ^$^*P* < 0.05 vs. LV-Rufy3 group

The correct Fig. 4 (caption):

**Fig. 4:**
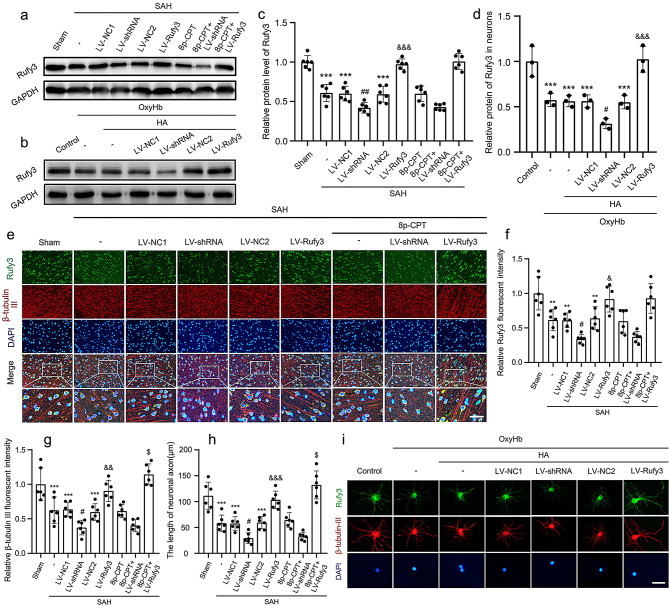
The protein expression levels of Rufy3 and the state of neuronal axon under LV-shRNA and LV-Rufy3 treatments after vivo and vitro SAH. **a** Representative bands of Rufy3 detected by western blot under 8p-CPT, LV-shRNA and LV-Rufy3 treatments following vivo SAH. **b** Representative bands of Rufy3 detected by western blot under LV-shRNA and LV-Rufy3 treatments following vitro SAH. **c**, **d** Quantitative analysis of Rufy3 in different groups following vivo and vitro SAH. The sham and control group were used as a control. e Double immunofluorescence analysis of Rufy3 (green, Alexa Fluor 488) and β-tubulin III (axon; red, Alexa Fluor 555); nuclei were stained with DAPI (blue). Scale bars = 32 μm. **f**, **g** Quantitative fluorescent intensity analysis of Rufy3 and β-tubulin III expressions in different groups. The sham group was used as the standard. **h** Quantitative analysis of the length of neuronal axon in different groups. i Double immunofluorescence of Rufy3 (green, Alexa Fluor 488) and β-tubulin III (axon; red, Alexa Fluor 555). Nuclei were stained with DAPI (blue). Scale bars = 100 μm. Data are shown as mean ± SEM (*n* = 6). ^**^P < 0.01, ^**^P < 0.001 vs. Sham group; ^*^P < 0.001 vs. Control group; ^#^*P* < 0.05, ^##^*P* < 0.01 vs. LV-NC1 groups; ^&^*P* < 0.05, ^&&^*P* < 0.01, ^&&&^*P* < 0.001 vs. LV-NC2 group; ^$^*P* < 0.05 vs. LV-Rufy3 group

The incorrect Fig. 6 (caption):

**Fig. 6:**
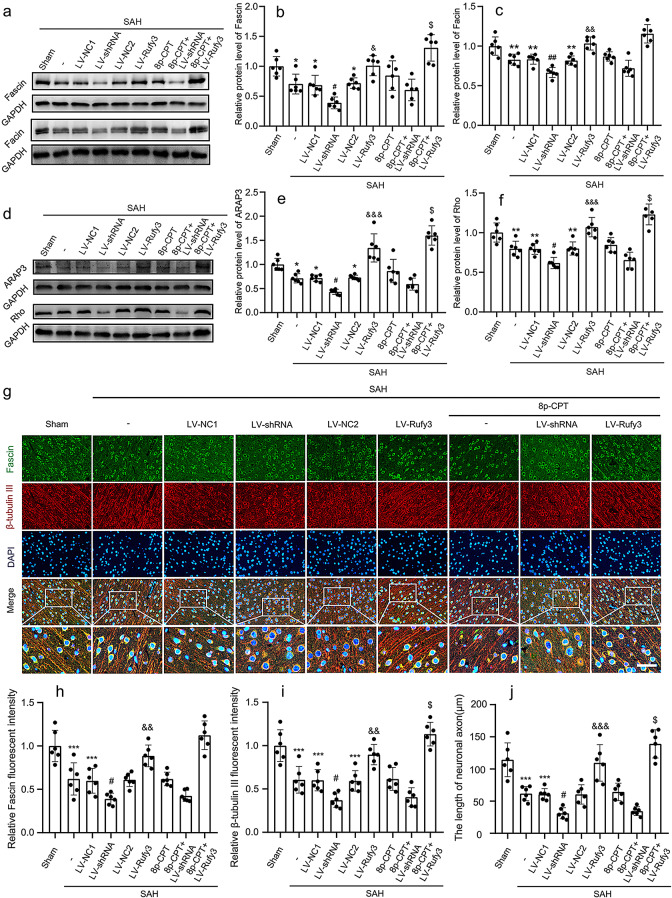
Effects of LV-shRNA and LV-Rufy3 on the Rap1/Arap3/Rho/Fascin signaling axis after experimental SAH. **a** Representative bands of Fascin and Facin expressions. **b**, **c** Quantitative analysis of Fascin and Facin. The sham group was used as control. **d** Representative bands of ARAP3 and Rho expressions. **e**, **f** Quantitative analysis of ARAP3 and Rho. The sham group was used as control. **g** Double immunofluorescence of Fascin (green, Alexa Fluor 488) and β-tubulin III (NeuN; red, Alexa Fluor 555); nuclei were stained with DAPI (blue). Scale bars = 40 μm. **h**, **i** Quantitative fluorescent intensity analysis of Rufy3 and β-tubulin III expressions in different groups. The sham group was used as the standard. **j** Quantitative analysis of the length of neuronal axons in different groups. ^*^P < 0.05, ^**^P < 0.01, ^***^P < 0.001 vs. Sham group; ^#^*P* < 0.05, ^##^*P* < 0.01 vs. LV-NC1 groups; ^&^*P* < 0.05, ^&&^*P* < 0.01, ^&&&^*P* < 0.001 vs. LV-NC2 group; ^$^*P* < 0.05 vs. LV-Rufy3 group

The correct Fig. 6:

**Fig. 6:**
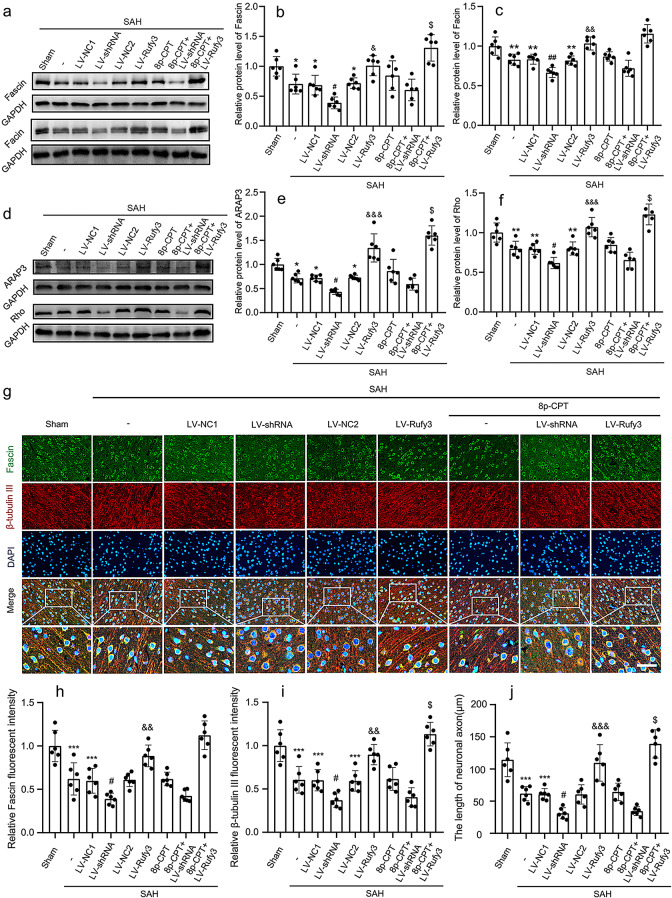
Effects of LV-shRNA and LV-Rufy3 on the Rap1/Arap3/Rho/Fascin signaling axis after experimental SAH. **a** Representative bands of Fascin and Facin expressions. **b**, **c** Quantitative analysis of Fascin and Facin. The sham group was used as control. **d** Representative bands of ARAP3 and Rho expressions. **e**, **f** Quantitative analysis of ARAP3 and Rho. The sham group was used as control. **g** Double immunofluorescence of Fascin (green, Alexa Fluor 488) and β-tubulin III (axon; red, Alexa Fluor 555); nuclei were stained with DAPI (blue). Scale bars = 40 μm. **h**, **i** Quantitative fluorescent intensity analysis of Rufy3 and β-tubulin III expressions in different groups. The sham group was used as the standard. **j** Quantitative analysis of the length of neuronal axons in different groups. ^*^P < 0.05, ^**^P < 0.01, ^***^P < 0.001 vs. Sham group; ^#^*P* < 0.05, ^##^*P* < 0.01 vs. LV-NC1 groups; ^&^*P* < 0.05, ^&&^*P* < 0.01, ^&&&^*P* < 0.001 vs. LV-NC2 group; ^$^*P* < 0.05 vs. LV-Rufy3 group

The incorrect Fig. 8:

**Fig. 8:**
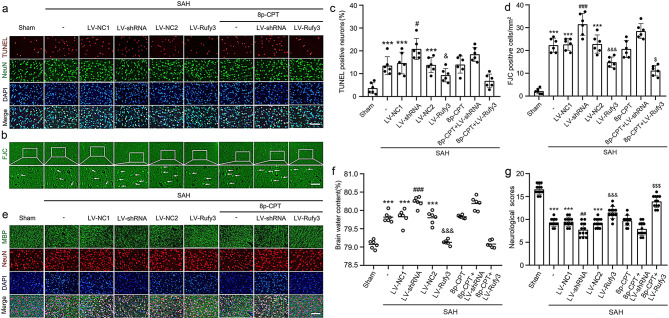
Effect of LV-shRNA and LV-Rufy3 on cortical cell apoptosis and degradation, brain edema, and neurological score after SAH. **a** Double immunofluorescence analysis of TUNEL staining (red, Alexa Fluor 555) and neuronal marker (NeuN; green, Alexa Fluor 488) was performed to assess neuronal apoptosis at 24 h after SAH. **b** Fluoro-Jade C staining (green) was performed to evaluate neuronal degeneration and arrows pointed to FJC-positive cells. **c** Quantitative analysis of apoptotic neuron percentage. **d** Quantitative analysis of Fluoro-Jade C positive cells/mm2 in brain sections in each group. **e** Double immunofluorescence of MBP (green, Alexa Fluor 488) and neuronal marker (NeuN; red, Alexa Fluor 555), and Rufy3 mainly located in the neurons. **f** Brain water content. **g** Neurological scoring. Scale bars = 100 μm. ^***^P < 0.001 vs. Sham group; ^#^*P* < 0.05, ^##^*P* < 0.01,^###^*P* < 0.001 vs. LV-NC1 groups; ^&^*P* < 0.05, ^&&&^*P* < 0.001 vs. LV-NC2 group; ^$^*P* < 0.05, ^$$$^*P* < 0.001 vs. LV-Rufy3 group

The correct Fig. 8:

**Fig. 8:**
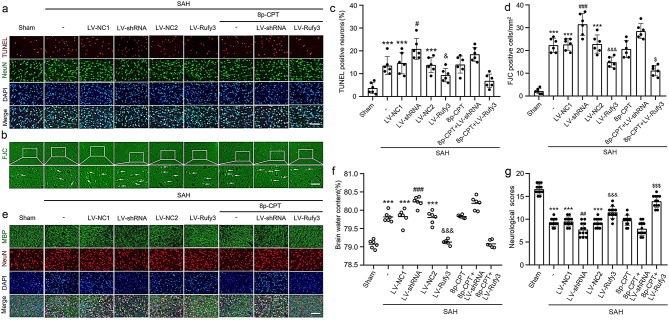
Effect of LV-shRNA and LV-Rufy3 on cortical cell apoptosis and degradation, brain edema, and neurological score after SAH. **a** Double immunofluorescence analysis of TUNEL staining (red, Alexa Fluor 555) and neuronal marker (NeuN; green, Alexa Fluor 488) was performed to assess neuronal apoptosis at 24 h after SAH. **b** Fluoro-Jade C staining (green) was performed to evaluate neuronal degeneration and arrows pointed to FJC-positive cells. **c** Quantitative analysis of apoptotic neuron percentage. **d** Quantitative analysis of Fluoro-Jade C positive cells/mm2 in brain sections in each group. **e** Double immunofluorescence of MBP (green, Alexa Fluor 488) and neuronal marker (NeuN; red, Alexa Fluor 555), and Rufy3 mainly located in the neurons. **f** Brain water content. **g** Neurological scoring. Scale bars = 100 μm. ^***^P < 0.001 vs. Sham group; ^#^*P* < 0.05, ^##^*P* < 0.01,^###^*P* < 0.001 vs. LV-NC1 groups; ^&^*P* < 0.05, ^&&&^*P* < 0.001 vs. LV-NC2 group; ^$^*P* < 0.05, ^$$$^*P* < 0.001 vs. LV-Rufy3 group

Figures 4, 6 and 8 have been updated above and the original article [1] has been corrected.
